# NTRC and thioredoxins *m*1/*m*2 underpin the light acclimation of plants on proteome and metabolome levels

**DOI:** 10.1093/plphys/kiad535

**Published:** 2023-10-07

**Authors:** Dejan Dziubek, Louis Poeker, Beata Siemitkowska, Alexander Graf, Giada Marino, Saleh Alseekh, Stéphanie Arrivault, Alisdair R Fernie, Ute Armbruster, Peter Geigenberger

**Affiliations:** Fakultät für Biologie, Ludwig-Maximilians-Universität München, Grosshaderner Str. 2-4, 82152 Martinsried, Germany; Fakultät für Biologie, Ludwig-Maximilians-Universität München, Grosshaderner Str. 2-4, 82152 Martinsried, Germany; Max-Planck-Institut für Molekulare Pflanzenphysiologie, Am Mühlenberg 1, 14476 Potsdam-Golm, Germany; Max-Planck-Institut für Molekulare Pflanzenphysiologie, Am Mühlenberg 1, 14476 Potsdam-Golm, Germany; Fakultät für Biologie, Ludwig-Maximilians-Universität München, Grosshaderner Str. 2-4, 82152 Martinsried, Germany; Max-Planck-Institut für Molekulare Pflanzenphysiologie, Am Mühlenberg 1, 14476 Potsdam-Golm, Germany; Departments of Metabolomics and Crop Quantitative Genetics, Center of Plant Systems Biology and Biotechnology, 4000 Plovdiv, Bulgari; Max-Planck-Institut für Molekulare Pflanzenphysiologie, Am Mühlenberg 1, 14476 Potsdam-Golm, Germany; Max-Planck-Institut für Molekulare Pflanzenphysiologie, Am Mühlenberg 1, 14476 Potsdam-Golm, Germany; Departments of Metabolomics and Crop Quantitative Genetics, Center of Plant Systems Biology and Biotechnology, 4000 Plovdiv, Bulgari; Max-Planck-Institut für Molekulare Pflanzenphysiologie, Am Mühlenberg 1, 14476 Potsdam-Golm, Germany; Institute of Molecular Photosynthesis, Heinrich Heine University Düsseldorf, Universitätsstrasse 1, 40225 Düsseldorf, Germany; CEPLAS—Cluster of Excellence on Plant Sciences, Heinrich Heine University Düsseldorf, Universitätsstrasse 1, 40225 Düsseldorf, Germany; Fakultät für Biologie, Ludwig-Maximilians-Universität München, Grosshaderner Str. 2-4, 82152 Martinsried, Germany

## Abstract

During photosynthesis, plants must manage strong fluctuations in light availability on different time scales, leading to long-term acclimation and short-term responses. However, little is known about the regulation and coordination of these processes and the modulators involved. In this study, we used proteomics, metabolomics, and reverse genetics to investigate how different light environmental factors, such as intensity or variability, affect long-term and short-term acclimation responses of Arabidopsis (*Arabidopsis thaliana*) and the importance of the chloroplast redox network in their regulation. In the wild type, high light, but not fluctuating light, led to large quantitative changes in the proteome and metabolome, accompanied by increased photosynthetic dynamics and plant growth. This finding supports light intensity as a stronger driver for acclimation than variability. Deficiencies in NADPH-thioredoxin reductase C (NTRC) or thioredoxins *m*1/*m*2, but not thioredoxin *f*1, almost completely suppressed the re-engineering of the proteome and metabolome, with both the induction of proteins involved in stress and redox responses and the repression of those involved in cytosolic and plastid protein synthesis and translation being strongly attenuated. Moreover, the correlations of protein or metabolite levels with light intensity were severely disturbed, suggesting a general defect in the light-dependent acclimation response, resulting in impaired photosynthetic dynamics. These results indicate a previously unknown role of NTRC and thioredoxins *m*1/*m*2 in modulating light acclimation at proteome and metabolome levels to control dynamic light responses. NTRC, but not thioredoxins *m*1/*m*2 or *f*1, also improves short-term photosynthetic responses by balancing the Calvin–Benson cycle in fluctuating light.

## Teaser

NTRC balances the Calvin–Benson cycle in fluctuating light and along with thioredoxins *m*1/*m*2 supports the re-engineering of the proteome and metabolome during acclimation to light intensity.

## Introduction

In nature, the level of irradiance can change dramatically with different dynamics ranging from rapid fluctuations due to shading by clouds or by neighboring plants to more long-term changes depending on the season, meteorological conditions, or time of day (Pearcy 1990; Reinhardt et al. 2010; Perez et al. 2016). This affects the amount of light energy available for photosynthesis and plant growth on different time scales. Plants have evolved short-term and long-term acclimation responses to manage these strong fluctuations in solar energy supply in a dynamic manner to optimize photosynthesis and growth in their natural environment.

Short-term acclimation includes various mechanisms operating within seconds to minutes to avoid transient imbalances in thylakoid energy conversion during rapid light fluctuations, which are sensed by changes in the photosynthetic electron transport (Dietz 2015; Kaiser et al. 2019). To cope with increased irradiation, photoprotective mechanisms are required to dissipate excessively absorbed light energy within seconds to minimize photo-oxidative damage and over-reduction of the photosystems (PS) I and II, while these mechanisms have to be rapidly reversed to increase the efficiency of photosynthetic energy conversion when light intensity is diminished. Protection against excessive light is linked to the proton gradient, which is generated across the thylakoid membrane during photosynthetic electron transport to synthesize ATP (Armbruster et al. 2017). The acidification of the thylakoid lumen inhibits the cytochrome (cyt) *b*_6_*f* complex and triggers the thermal dissipation of excess light energy at PSII by nonphotochemical quenching (NPQ), which involves reversible interactions between PsbS and the light-harvesting complex (LHC) II regulated by changes in violaxanthin levels (Müller et al. 2001). Excess electrons accumulating at the acceptor side of PSI are transferred back to plastoquinone (PQ) by cyclic electron transport, which pumps protons into the thylakoid lumen via proton gradient regulation (PGR) protein PGR5/PGRL1 and NADH dehydrogenase-like (NDH) dependent pathways to synthesize ATP without producing NADPH and to promote NPQ (Shikanai 2020; Yadav et al. 2020). In addition, antioxidative processes operate in the chloroplast stroma to detoxify harmful reactive oxygen species (ROS), which are generated by transferring excess photosynthetic electrons to O_2_ (Asada 1999).

In the long-term, dynamic acclimation to changing light intensities also involves down-stream processes in metabolism and gene expression, leading to a reprograming of the proteome and metabolome in a time frame of hours to several days, involving retrograde signaling from the chloroplast to the nucleus (Walters 2005; Dietz 2015; Morales and Kaiser 2020). In Arabidopsis (*Arabidopsis thaliana*), dynamic acclimation to constant high light (HL) was shown to involve both large changes in the proteome with an increased abundance of proteins involved in photosynthetic electron transport and carbon metabolism (Miller et al. 2017) and changes in the metabolome, characterized by increased levels of photosynthetic end-products, such as sugars, organic acids and amino acids (Garcia-Molina et al. 2020), which both took several days to develop. These responses are important to maximize photosynthetic capacity and increase plant growth under longer periods of elevated light intensity. In comparison to this, dynamic acclimation to fluctuating light (FL) within a similar time frame involved only moderate but widespread alterations in protein abundance, which included enhanced accumulation of proteins involved in photoprotection, cyclic electron flow (CEF) around PSI, photorespiration, and glycolysis, while specific glutathione transferases and proteins involved in translation and chlorophyll biosynthesis were decreased (Niedermaier et al. 2020). These responses are important to increase the capacity of photoprotection during longer periods of FL treatments, while there is a trade off in plant growth. However, studies are missing to analyze the changes in the metabolome in FL and to compare FL and HL in the same experiments.

A central mechanism to regulate light acclimation responses involves redox signals (Walters 2005; Bräutigam et al. 2009; Dietz 2015), with thioredoxins (Trx) playing a crucial role in balancing stromal metabolic processes (MF) with the light-driven electron transport to avoid transient imbalances in NADPH/NADP^+^ redox and ATP/ADP energy states during rapid fluctuations in light intensity (Nikkanen and Rintamaki 2014; Geigenberger et al. 2017). Thioredoxins comprise a small family of proteins with protein disulfide reductase activity that catalyze Cys-based post-translational modifications of target proteins to modulate their functions (Meyer et al. 2012). In the chloroplast, Trxs is reduced in response to light by sequential transfer of photosynthetic electrons from ferredoxin (Fdx) via Fdx:Trx reductase (FTR) to different Trx isoforms grouped into 5 subtypes (Trxs *f*1-2, *m*1-4, *x*, *y*1-2 and *z*), which activate specific sets of target proteins by reducing their regulatory disulfides within a time-frame of seconds to minutes (Geigenberger et al. 2017; Thormählen et al. 2019; Zaffagnini et al. 2019). Thioredoxin *f* comprises 22% of total chloroplast Trxs (Okegawa and Motohashi 2015). There is a role of *f-*type Trx in short-term activation of the Calvin–Benson cycle (CBC) and starch synthesis during rapid dark-light transitions, with Trx *f* deficiency leading to retarded redox-activation of fructose-1,6-bisphosphatase (FBPase), Rubisco activase and ADP-glucose (ADPG) pyrophosphorylase (AGPase), resulting in a delayed increase in photosynthetic efficiency and a transient increase in NPQ in Arabidopsis *trxf1* (Thormählen et al. 2013; Thormählen et al. 2015) and *trxf1f2* mutants (Yoshida et al. 2015; Naranjo et al. 2016a). Light induction of photosynthesis was also found to be controlled by Trx *m*4, which regulates cyclic electron transport around PSI via interaction with PGRL1 (Courteille et al. 2013; Okegawa and Motohashi 2020), thereby modulating the transient rise in NPQ and the induction of photosynthetic efficiency during dark-light transitions by a different mechanism than *f*-type Trxs, although both mechanisms were additive (Okegawa et al. 2020). While *trxf1* and *trxf1f2* mutants showed retarded growth only in very short days or extremely low light (LL) (Thormählen et al. 2015; Naranjo et al. 2016a), *trxf1trxf2pgrl1* triple mutants revealed growth defects already in normal long-day conditions (Okegawa et al. 2020). While this shows that Trxs *f*1, *f*2, and *m*4 are involved in regulating photosynthetic performance during dark-light transitions, little is known about their roles in the acclimation of plants to FL intensities.

By contrast, Trxs *m*1 and *m*2—which comprise more than 50% of all chloroplast Trxs (Okegawa and Motohashi 2015)—showed no substantial role during dark-light transitions. Both *m*-type Trxs were found to affect photosynthetic performance during acclimation in FL environments, with combined deficiencies in Trxs *m*1 and *m*2 in Arabidopsis *trxm1m2* mutants leading to lower photosynthetic efficiency in the HL periods but surprisingly higher photosynthetic efficiency in the LL phases of FL (Thormählen et al. 2017). It was suggested that the lower photosynthetic efficiency in the HL peaks is due to a reduced capacity of the *trxm1m2* mutants in rapid redox-activation of NADP-malate dehydrogenase (NADP-MDH) involved in the export of reducing power from the chloroplast (Thormählen et al. 2017). While this did not affect plant growth in the *trxm1m2* mutants (Thormählen et al. 2017), Arabidopsis mutants whose NADP-MDH lacks one of its redox switches exhibited severe growth retardations in FL environments (Yokochi et al. 2021). Since plants were exposed to FL for several days in these studies, Trx *m*1/*m*2 deficiency may also have led to more long-term effects on photosynthetic efficiency in addition to the short-term restriction in thiol-disulfide regulation of NADP-MDH.

In addition to the light-dependent Fdx/Trx system, chloroplasts contain an NADPH-dependent Trx reductase (NTRC), which receives its reducing potential from NADPH and provides electrons to target proteins via its own tethered Trx domain (Serrato et al. 2004). Recent biochemical and genetic studies showed that both thiol-redox systems are interlinked, with NTRC being involved in the light-dependent regulation of photosynthetic metabolism similar to the Fdx/Trx system (Thormählen et al. 2015; Yoshida and Hisabori 2016). While NTRC is not able to control the reduction state of CBC enzymes directly (Ojeda et al. 2017), it uses NADPH to reduce 2-Cys-peroxiredoxins (2Cys-Prx), which indirectly modulates the reduction state of Trx-regulated target enzymes (Perez-Ruiz et al. 2017). NTRC was found to be specifically important during dark-light transitions (Thormählen et al. 2017) and under limiting light intensities (Carrillo et al. 2016; Naranjo et al. 2016b), where NTRC allows efficient redox activation of the CBC (CBC) and proton-coupled ATP synthase, leading to lower acidification of the thylakoid lumen and lower NPQ, which results in more efficient utilization of light energy for photosynthesis and growth. The decrease in NPQ may also be attributable to NTRC inhibiting cyclic electron transport via redox regulation of PGRL1 (Naranjo et al. 2021), while the importance of this mechanism to explain the role of NTRC in dark-light transition and LL acclimation remains unclear. In addition to this, genetic evidence was provided that NTRC plays a central role in dynamic acclimation of photosynthesis in FL regimes (Carrillo et al. 2016; Thormählen et al. 2017) to ensure the full range of dynamic responses of NPQ and PQ reduction state following the alternating LL and HL periods of FL, to optimize photosynthesis and maintain plant growth under these conditions (Thormählen et al. 2017). Since, in this study, plants were exposed to FL for several days, NTRC deficiency may also have led to long-term effects on photosynthesis that may explain the restrictions in the short-term regulation of NPQ and chloroplast metabolism.

While Trxs act on a time-scale of seconds to minutes to allow rapid-light activation of chloroplast enzymes and stromal metabolism during dark-light transitions (Zimmer et al. 2021), little is known about their roles in regulating more long-term light acclimation responses of plants within a time-scale of several days. In this study, we used proteomics, metabolomics, and physiological analyses combined with reverse genetic approaches to investigate the roles of Trx *f*1, Trxs *m*1/*m*2, and NTRC in dynamic acclimation to FL, HL, and LL. Our results show that NTRC is important to improve photosynthetic acclimation in HL and FL via long-term effects operating within a time frame of several days and resulting in optimized growth rates. This is attributable to NTRC being indispensable in allowing large-scale re-engineering of the proteome and metabolome specifically during HL acclimation, which involves translational regulation. In addition, Trxs *m*1 and *m*2 are involved in re-engineering the proteome and metabolome during HL, although with less impact on photosynthetic performance and growth under these conditions. This contrasts with Trx *f*1 having only minor effects on light acclimation responses, although there is a slight involvement in FL responses of the proteome.

## Results

### Dynamic acclimation of plant growth in HL and FL is impaired in the *ntrc* mutant

**Figure 1.**
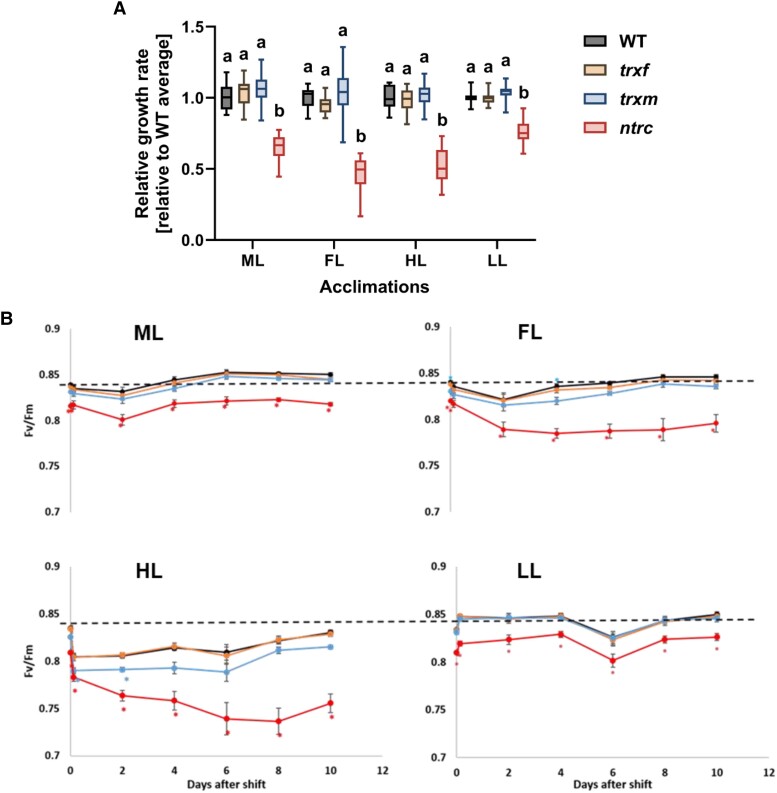
Growth and photosynthetic acclimation of WT, *trxf1*, *trxm1m2,* and *ntrc* mutants in different light intensities and in FL. WT (black), *trxf1* (*trxf,* yellow), *trxm1m2* (*trxm,* blue), and *ntrc* (red) mutants were grown in 12h-day length at ML intensity (250 *µ*mol photons m^−2^ s^−1^) for 3 weeks and then kept at this light intensity or shifted to either LL (90 *µ*mol photons m^−2^ s^−1^), HL (900 *µ*mol photons m^−2^ s^−1^) or fluctuating LL and HL (FL, 4 min LL, 1 min HL, average light intensity: ∼250 *µ*mol photons m^−2^ s^−1^) for 10 days (d). **A)** Rosette-growth rates of the mutants after the shift to the respective light conditions represented as boxplots relative to WT level. Measurements of rosette-leaf areas were performed every 2 d for 10 d total. The rate was calculated from curve fitting as mean value of all time points. The line in the middle of the boxplot is the median. The bottom and top of the boxplots are the 25th (Q1) and 75th (Q3) percentiles, respectively, and define the interquartile range (IQR; Q3-Q1). The whiskers extend to the maximum (Q3 + 1.5 * IQR) and the minimum (Q1–1.5 * IQR). Results are the mean, with *n* = 16 to 18 biological replicates per genotype and condition. **B)** Maximum quantum efficiency of photosystem II (Fv/Fm) in the different genotypes growing in ML and before and after the shift for 2 h, 2 d, 4 d, 6 d, 8 d, and 10 d to FL, HL or LL. The dashed line indicates the dark-adapted WT level in ML. Results are the mean ± SE, with *n* = 23 to 28 biological replicates per genotype and condition. Different letters (A) and asterisks (B) indicate where the values of mutants are significantly different from the respective WT (1-way ANOVA with a post hoc Tukey test; *P* < 0.05).

In the present study, Arabidopsis mutants deficient in Trx *f*1, Trxs *m*1/*m*2, or NTRC were grown together with wild type (WT) for 3 weeks in medium light (ML, 250 µE) before plants were shifted to LL (90 µE), HL (900 µE) or FL (1 min HL, 4 min LL) or stayed at ML for another 10 days. Light-emitting diode (LED) illumination was used to avoid heat development in HL conditions. We first determined the growth rate of plants by analyzing the increase in leaf area starting from 2 days after the shift. Inspection of Supplemental Fig. S1A shows that light shifts affected the rosette growth rate of WT plants, leading to a decrease in FL, while there was an increase in HL when compared to ML. This confirms previous studies, where FL led to a decrease (Kaiser et al. 2019; Morales and Kaiser 2020; von Bismarck et al. 2023), while HL led to a stimulation of plant growth (Thormählen et al. 2015; von Bismarck et al. 2023). Compared to WT, the rosette growth rate of the *ntrc* mutant was significantly diminished, particularly in FL and HL (Fig. 1A). The decreases in the relative growth rates of the *ntrc* mutant after the shifts from ML to FL and ML to HL (Fig. 1A) were shown to be significant, with *P* < 0.0001 (ML vs. FL) and *P* = 0.0002 (ML vs. HL), respectively (2-way ANOVA, Dunnett's multiple comparison test). This shows growth rates of the *ntrc* mutant are particularly sensitive to HL and FL shifts, while *trxf1* and *trxm1m2* mutants revealed WT-like growth.

### Recovery of photosynthetic efficiency during long-term acclimation in HL and FL is impaired in *ntrc* and *trxm1m2* mutants

In the case of chloroplast-localized processes, changes in growth rates may be due to alterations in photosynthesis. To investigate whether the shifts from ML to FL, HL, or LL affect the maximum quantum efficiency of PSII in the different genotypes, we monitored Fv/Fm at different time points between 2 h and 10 days after the light shift, which represents a photosynthetic parameter that is sensitive to oxidative stress (Fig. 1B). In the WT, the Fv/Fm value initially decreased in response to a shift from ML to FL or HL, but fully recovered after 10 days of acclimation, confirming previous studies (Thormählen et al. 2017). In comparison to WT, the *ntrc* mutant exhibited a similar initial decrease within the first 2 h after the shift from ML to FL or HL; however, this was followed by a much steeper and more progressive decline in Fv/Fm in both conditions during the following days. This included a progressive drop in Fv/Fm from 0.82 to 0.77 within 4 days in FL and from 0.81 to 0.72 within 8 days in HL, with no substantial subsequent recovery. With respect to WT, the *trxm1m2* mutant also showed a stronger decrease in Fv/Fm and a delayed recovery, specifically after the HL shift, while the *trxf1* mutant was similar to WT in all light conditions. These data suggest that NTRC is indispensable for long-term acclimation of photosynthesis in both HL and FL, while Trxs *m*1/*m*2 are important to facilitate photosynthetic acclimation, specifically in HL.

### NTRC deficiency impairs photosynthetic dynamics in response to long-term acclimation in HL and FL

To further investigate how long-term light acclimation shapes the dynamics of photosynthesis in the different genotypes, plants acclimated to the respective light regimes for seven days were subsequently exposed for 20 min to either a step-wise increase in light intensities (six different light intensities ranging from 0 to 500 µE) or FL (alternating periods of 4 min LL and 1 min HL) to analyze time-resolved changes in chlorophyll fluorescence. In the WT, short-term exposure to step-wise increasing light intensities led to a steady increase in NPQ (nonphotochemical quenching) and a saturating response in photosynthetic electron transfer rate (ETR) (Supplemental Fig. S1B), the extent being dependent on light acclimation conditions, with HL acclimation leading to a diminished increase in NPQ and a stronger increase in ETR, and FL acclimation leading to opposite responses. This indicates that in the WT, long-term acclimation to HL or FL differentially affects dynamics in NPQ and ETR in response to a short-term increase in light intensity, which is in confirmation of previous studies (von Bismarck et al. 2023). In comparison to WT, the *ntrc* mutant exhibited an exaggerated increase in NPQ, while photosynthetic ETR was strongly restricted in response to short-term increases in light intensity, specifically in HL- and FL-acclimated plants. Compared to this, the *trxf1* and *trxm1m2* mutants showed WT responses, except for a slight decrease in ETR (and increase in NPQ) in HL-acclimated plants subjected to short-term HL.

**Figure 2.**
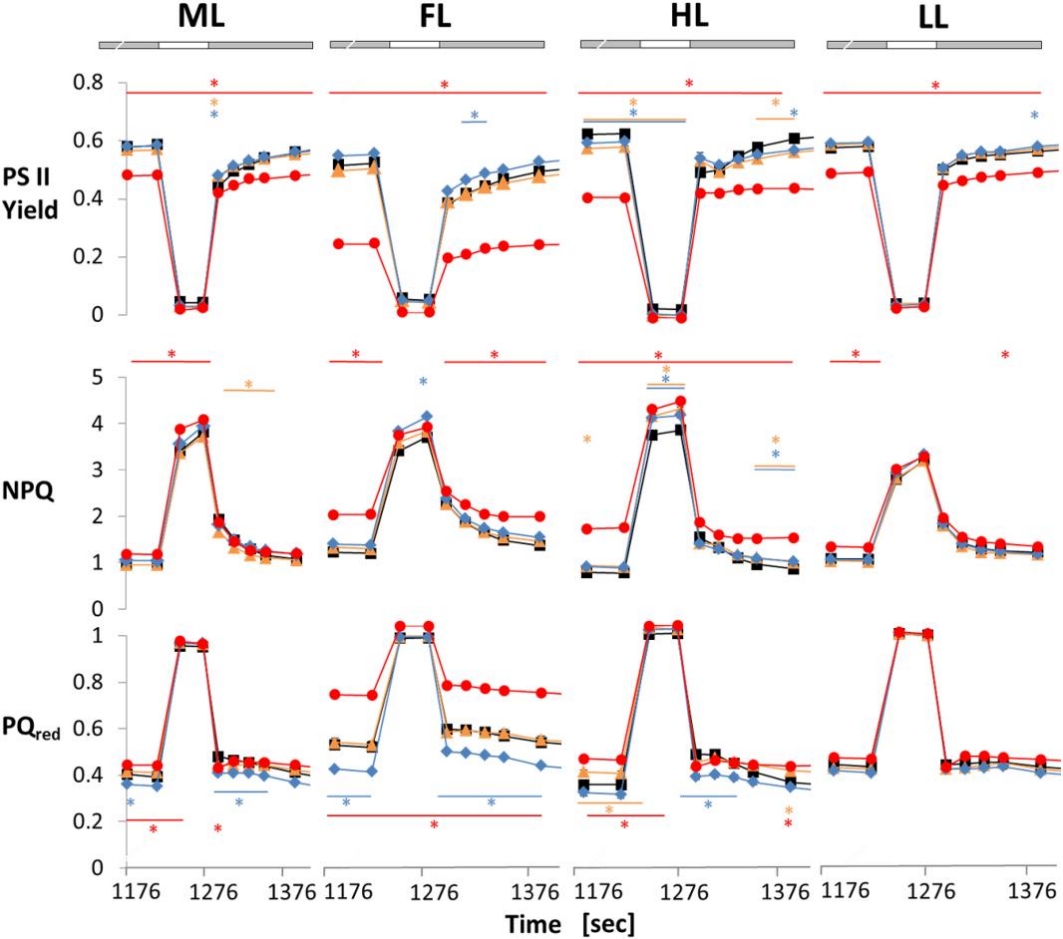
Light acclimation effects on photosynthetic dynamics are restricted in the *ntrc* mutant. WT (black), *trxf1* (*trxf,* yellow), *trxm1m2* (*trxm,* blue), and *ntrc* (red) mutants were grown in 12 h-day length at ML intensity (250 *µ*mol photons m^−2^ s^−1^) for 3 weeks and then kept at this light intensity or shifted to either LL (90 *µ*mol photons m^−2^ s^−1^), HL (900 *µ*mol photons m^−2^ s^−1^) or fluctuating LL and HL (FL, 4 min LL, 1 min HL, average light intensity: ∼250 *µ*mol photons m^−2^ s^−1^) for 7 d. After 30 min of dark-adaptation, plants were subsequently transferred for 20 min to short-term FL (see above) to analyze short-term dynamics of different chlorophyll fluorescence parameters using PAM fluorometry: photosystem II quantum efficiency (PSII yield; top row), NPQ (middle row) and PQ pool reduction state (PQ_red_, measured as 1-qL; bottom row). An increase in PQ_red_ is indicative of decreased PSII acceptor availability. Results are the mean ± SE, *n* = 6 to 12 biological replicates. Asterisks above and below traces mark were parameters were significantly different relative to WT. Significant changes, relative to WT, within one condition were evaluated by using a one-way ANOVA with a post hoc Tukey test (*P* < 0.05). The gray bars indicate the LL, the white bars the HL phases of FL.

When WT plants were exposed to 20 min short-term FL, alternating light intensities led to high NPQ in the face of low PSII acceptor availability and PSII quantum efficiencies in the HL periods and reverse effects in the LL periods (Fig. 2). Interestingly, the dynamics of these short-term changes were dependent on light acclimation conditions, with long-term acclimation to HL leading to increased, and FL acclimation leading to a decreased dynamic range of these parameters during the fluctuation when compared to ML. This indicates that in the WT, long-term acclimation to HL improves photosynthetic dynamics in short-term FL, while photosynthetic dynamics diminish during long-term acclimation in FL. In comparison to WT, the *ntrc* mutant exhibited a restriction of the dynamic responses in these photosynthetic parameters, specifically in the LL periods of FL, confirming previous studies (Thormählen et al. 2017). This involved strong attenuations in the recovery of PSII quantum efficiency and in the relaxations of NPQ and reduced PQ_red_ in the LL phases of short-term FL exposure (Fig. 2). These restrictions in photosynthetic dynamics in response to NTRC deficiency were much more severe in plants acclimated to HL or FL, compared to LL or ML (Fig. 2). This shows that in the *ntrc* mutant, the FL-induced impairment of photosynthetic dynamics occurred within a time-frame of several days, rather than minutes. Crucially, this attenuating effect was not seen in ML-grown plants subjected to short-term FL. Compared to this, the *trxf1* mutant showed WT responses, while the *trxm1m2* mutant showed a behavior slightly different to WT with improved photosynthetic efficiency and decreased PQ_red_ in the LL phases of short-term FL exposure, confirming previous studies (Thormählen et al. 2017). This response of the *trxm1m2* mutant was more strongly expressed in plants acclimated to FL but was also visible after acclimation to the other light conditions, indicating that it is mainly a short-term effect that occurs within minutes which is less affected by long-term acclimation conditions (Fig. 2).

*In summary*, these results indicate that long-term acclimation in HL or FL shapes the dynamics of photosynthesis, with NTRC being important to optimize PSII quantum efficiency and dynamic responses of NPQ under these conditions.

### Global proteome changes fall into 2 different clusters related to translation and stress responses, revealing large-scale alterations in protein abundance in response to HL acclimation that are strongly repressed in the *ntrc* and *trxm1m2* mutants

The results so far showed that in response to long-term acclimation in HL or FL, there were changes in photosynthetic dynamics, which were strongly attenuated in the *ntrc* mutant. While short-term light acclimation in the time-frame of seconds to minutes will involve post-translational regulatory mechanisms acting on existing proteins, long-term acclimation in the time-frame of days will also involve de-novo synthesis and degradation of specific proteins to allow modifications in the photosynthetic machinery (Walters 2005). To investigate the acclimation responses of the proteome in WT, *trxf1*, *trxm1m2*, and *ntrc* mutants, we harvested plant rosettes seven days after the shifts from ML to FL, HL, or LL by freezing into liquid N_2_ at 6 h into the photoperiod to be used for quantitative label-free proteomics analysis. We obtained 2,758 peptide hits (respective 3,970 unique peptides) translated to 2,964 unique AGI locus identifiers for down-stream analyses (Arabidopsis Genome Initiative 2000). The complete proteomic data set, including mean values and statistics, is summarized in Supplemental Table S1 (see Supplemental). To get an overview of the behavior of the global proteome in the different light conditions and genotypes, we used four different methods to visualize the data.

**Figure 3.**
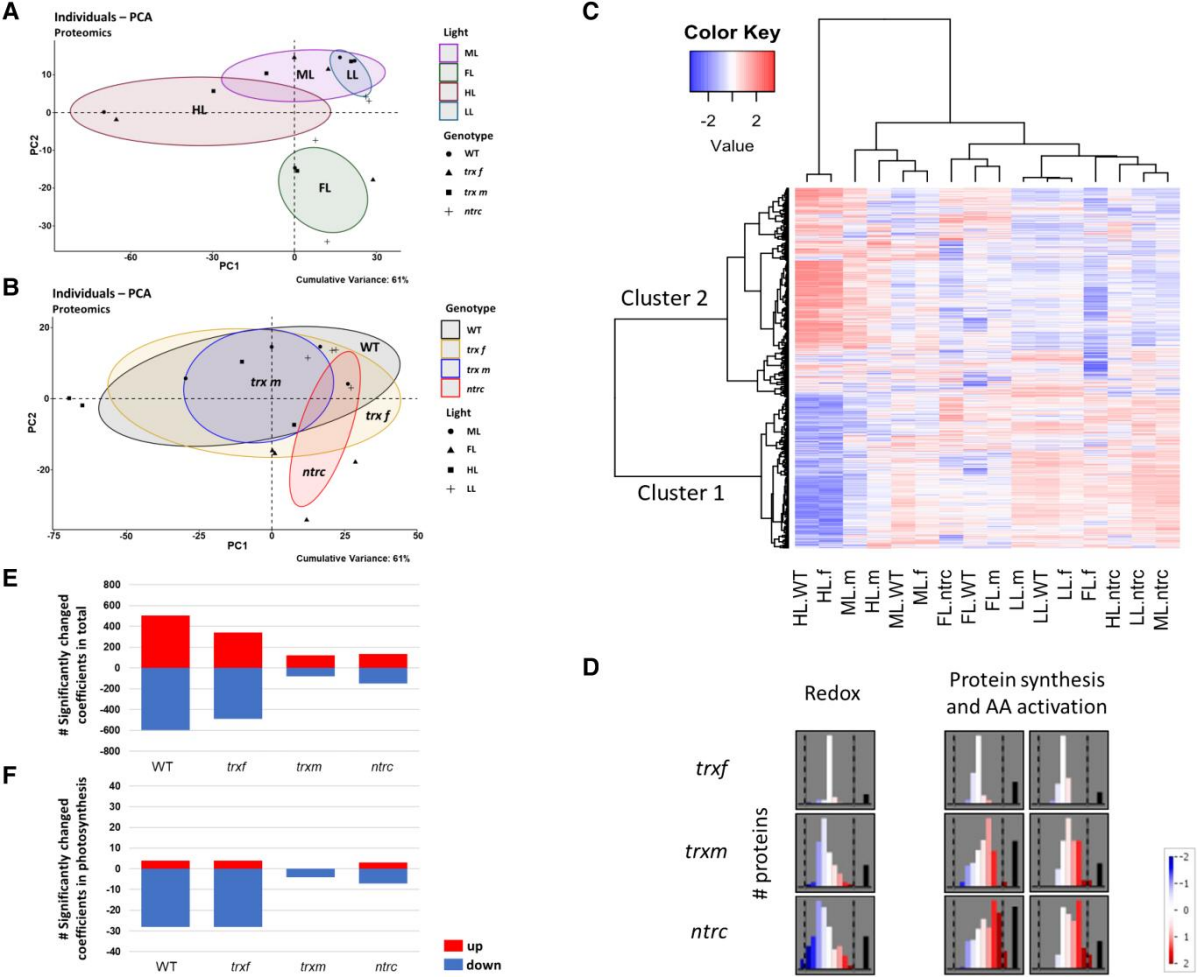
Light-intensity dependent changes in the global proteome are strongly attenuated in both *ntrc* and *trxm1m2* mutants. WT, *trxf1* (*trxf*), *trxm1m2* (*trxm*), and *ntrc* mutants were grown for 7 days in different acclimation conditions (ML, LL, HL, or FL, for details see legend of Fig. 2), before rosettes were sampled 6 h into the photoperiod to analyze proteomic changes using principle component analysis (A, B), cluster analysis (C), MapMan software (D), and linear regression analysis (E, F). **A, B)** Principle component analysis (PCA) of light-dependent (A) and genotypic changes (B) of the proteome. The cumulative variance explained by PC1 and PC2 is 61%. Plots were generated with R package “factoextra” and “FactoMineR”. **C)** Representative heat map of standardized protein abundances. Only valid peptide hits common in all light regimes were incorporated. The heat map shows z-scored changes in protein abundance, with blue color indicating down-regulation, while red color indicating up-regulation with respect to the mean of all samples. The plot is separated into two clusters. The row clustering (proteins abundance) outlines the overall adjustment, the column clustering indicates the relation between different samples. Clustering distance: correlation; clustering method: Ward's method. Heat map was generated with R package “gplots” (Warnes et al. 2015). (f) *trxf1*; (m) *trxm1m2*. **D)** Summary of the preferential changes in protein abundance in the major functional MapMan categories “redox” and “protein synthesis & AA activation” during HL acclimation in different mutants, compared to WT. Relative abundances are shown as log2-fold changes relative to WT ranging from −2 (blue) to 2 (red). White: No change. Black bar: Amount of proteins not expressed in this bin. Graphs were generated with MapMan software (see also Supplemental Figs. S3 to S5 in the supplement for more details). **E, F)** Quantitative correlation of global protein levels and light intensity. Linear regression analysis of proteins was performed using acclimation-light intensity (photon flux density in µE) as independent variable. The number of significantly changed regression coefficients (*P* < 0.05) in the total proteome (E) and in the photosynthetic proteome based on the chloroplast genome (F) is shown. Raw data, see Supplemental Table S1.

We first analyzed the response of the proteome using principal component analysis (PCA), which is a dimension-reduction technique that gives information about which different genotypes or light conditions are closely related or separated with respect to the pattern in protein abundance. The PCA of the proteome data exhibited large changes in the protein pattern in response to shifts from ML to HL or FL, while changes were minor after a shift from ML to LL (Fig. 3A). With respect to the different genotypes, the protein profile of the *ntrc* mutant was largely separated from WT, while those of *trxf1* and *trxm1m2* mutants were close to WT, with *ntrc* and *trxm1m2* showing a smaller variance than the other genotypes (Fig. 3B).

Secondly, global changes in protein abundance were displayed within a heatmap (Fig. 3C). Using cluster analysis of the z-scored data, we identified two different protein clusters showing differential protein patterns with respect to light conditions and genotypes. In the WT, cluster 1 mainly represented proteins that showed, on average, high levels in LL, ML, and FL but very low levels in HL, while cluster 2 proteins showed the opposite with high levels in HL conditions. With respect to the different genotypes, the protein pattern of the *trxf1* mutant was close to WT in all light conditions, while those of the *ntrc* and *trxm1m2* mutant were clearly separated from WT. Specifically, NTRC deficiency almost completely prevented the HL-induced shifts in the protein pattern, with cluster 1 and cluster 2 proteins remaining at high and low levels, respectively, even under HL conditions. To investigate the most significant functional categories of the proteins belonging to clusters 1 and 2 with respect to non-redundant gene ontology (GO) terms according to REVIGO, the AgriGO v2.0 web-based tool and database for GO were used for statistical analysis. Table 1 lists the major functional categories associated with clusters 1 and 2, showing that the changes in the proteome linked to HL acclimation in the WT are preferentially related to translation (cluster 1) and stress/abiotic stimulus responses (cluster 2), being repressed and induced, respectively. These changes were nearly completely abolished in *ntrc* and strongly attenuated in *trxm1m2* mutants (Fig. 3C).

**Table 1.** Significant functional categories of proteomic changes associated with clusters 1 and 2

Cluster	Description	−log10 *P*-value
	**Biological process**	
1	Translation	17.60
1	Gene expression	13.51
1	Protein metabolic process	8.26
1	Cellular macromolecule metabolic process	7.32
1	Biosynthetic process	6.66
1	Macromolecule metabolic process	5.39
1	Nitrogen compound metabolic process	4.77
1	Primary metabolic process	2.55
1	Cellular metabolic process	2.15
1	Transport	2.13
1	Macromolecule modification	1.80
1	Localization	1.68
1	Cell communication	1.47
1	Cellular process	1.38
2	Response to abiotic stimulus	6.39
2	Response to stress	6.11
2	Response to stimulus	5.11
2	Response to endogenous stimulus	4.39
2	Catabolic process	3.82
2	Carbohydrate metabolic process	2.28
2	Homeostatic process	2.20
2	Response to biotic stimulus	2.14
2	Multi-organism process	1.92
2	Secondary metabolic process	1.89
2	Precursor metabolites and energy generation	1.64
2	Regulation of biological quality	1.60
	**Cellular component**	
1	Intracellular ribonucleoprotein complex	10.00
1	Nonmembrane-bounded organelle	9.52
1	Macromolecular complex	5.33
1	Nucleolus	4.21
1	Nuclear part	3.85
1	Endosome	1.92
1	Membrane-enclosed lumen	1.66
1	Peroxisome	1.40
1	Microbody	1.40
2	Thylakoid	5.19
2	Extracellular region	4.22
	**Molecular function**	
1	Structural molecule activity	11.57
1	Nucleic acid binding	9.07
1	RNA binding	8.38
1	Nucleotide binding	3.80
1	Nucleoside-triphosphatase activity	3.62
1	Hydrolase activity, acting on acid anhydrides	3.04
1	Transporter activity	2.20
1	Translation factor activity, RNA binding	2.05
2	Transcription factor activity, sequence-specific DNA binding	2.09

Table of significant GO terms sorted by −log 10 *P*-values regarding the proteomic changes shown in Fig. 3C. A list of complete GO terms was created with “agriGO” and sent to “REVIGO” to remove redundant terms (Supek et al. 2011; Tian et al. 2017). Clusters 1 and 2 are corresponding to Fig. 3C.

Thirdly, MapMan software (Thimm et al. 2004) was used to visualize preferential changes in protein abundance in different functional categories (Supplemental Figs. S2 to S6). Supplemental Fig. S2 provides an overview of the direction and extent of the changes in protein levels in selected MapMan bins related to different cell functions, comparing light shifts from ML to FL, HL, or LL in the WT. With respect to the HL shift, the data in Supplemental Fig. S2E showed a strong and preferential increase in proteins involved in stress responses and redox processes, while proteins involved in protein synthesis, amino acid (AA) activation, RNA processing, and transport showed the opposite effect. Supplemental Figs. S2, F and G show the HL-induced changes in these functional categories in more detail, indicating a preferential increase in proteins involved in heat stress and redox processes, such as Trxs, enzymes of ascorbate/glutathione metabolism, glutaredoxins, peroxiredoxins, and dismutases/catalases (Supplemental Fig. S2F), while proteins involved in RNA processing, RNA transcription, AA activation, ribosomal structure, ribosome biogenesis, and protein synthesis (initiation, elongation, and release) were preferentially decreased (Supplemental Fig. S2G). The HL shift also led to a preferential increase in proteins involved in CBC and flavonoid metabolism, while proteins involved in photosynthetic light reactions and terpene metabolism, specifically carotenoid metabolism and zeaxanthin synthesis (important for NPQ formation), were decreased (Supplemental Figs. S2H and S6A). In comparison to these strong trends in functional changes in HL, the shifts from ML to FL (Supplemental Fig. S2, A to D) or LL (Supplemental Fig. S2, I to L) only led to very minor effects in protein abundance in the WT. Compared to WT, the largest differences in these functional categories were found in the *ntrc* (Supplemental Figs. S3 and S6B) and *trxm1m2* mutants (Supplemental Figs. S4 and S6C), specifically under HL conditions (Supplemental Figs. S3, E to H, S4, E to H and S6, B and C), where the differences were opposite to the HL-induced WT responses (compare with Supplemental Figs. S2 and S6A), while the *trxf1* mutant was similar to WT (Supplemental Figs. S5 and S6D). This shows that the HL-responsive changes in protein abundance observed in the WT are strongly repressed in both *trxm1m2* and *ntrc* mutants. The effect of the different mutants on the preferential changes in protein abundance of the major categories “redox processes” and “protein synthesis & AA activation” during HL acclimation is summarized in Fig. 3D, showing that both *trxm1m2* and *ntrc* mutants leading to strong alterations compared to WT, while *trxf1* showed WT-like responses. While genotypic effects in FL were relatively minor, Trx *f*1 deficiency led to substantial differences in protein abundance under this condition, with proteins involved in abiotic stress responses and redox regulation showing a tendency to be higher compared to WT level (Supplemental Fig. S5, A to D). This suggests *f*- and *m*-type Trxs have different roles in the acclimation of the proteome in different light conditions.

Fourth, to investigate the light-dependent and genotypic variations in global protein abundance in more detail, we used statistics to analyze the number of differentially expressed proteins (DEPs) and their gene GO term enrichments according to REVIGO to identify the most significantly changed functional categories via the AgriGO v2.0 web-based tool. With respect to light-dependent variations in the WT, the number of significantly changed proteins was much higher after the shift from ML to HL (600) compared to the shifts to LL (201) or FL (35). This shows that HL acclimation involves more DEPs than acclimation to LL or FL, with a majority being localized outside the chloroplast (Supplemental Fig. S7). Focusing on WT DEPs that were increased in HL, the most significantly changed categories involved responses to stress and diverse stimuli, cell redox homeostasis and carbohydrate metabolism with respect to biological process (BP), metal ion binding and antioxidant activity with respect to molecular function (MF), and association with apoplast and chloroplast stroma with respect to cellular component (CC), while those that were decreased involved translation, ribosome biogenesis, macromolecule biosynthetic processes and gene expression (BP), structural constituents of ribosomes and nucleic acid binding (MF), and association with cytosolic ribosomal subunits (CC) (Supplemental Table S2). Increased DEPs involved proteins associated with antioxidative function (ascorbate and glutathione metabolism, peroxiredoxins, catalase, and peroxiredoxins), Trxs (including Trx *m*4, which activates CEF), metabolism (CEF including PGR5 and NDH complex, CBC, photorespiration, starch degradation, glycolysis, TCA cycle, respiration, flavonoid, and phenylpropanoid synthesis), protein targeting and signaling (Supplemental Table S1). In contrast to this, decreased DEPs involved proteins associated with protein synthesis (translation), degradation, and modification mainly localized in the cytosol, and a set of proteins associated with photosynthetic light reactions in the chloroplast, including PsbS and zeaxanthin epoxidase involved in NPQ generation (Supplemental Table S1). In response to the LL shift, the most significantly increased GO terms in the WT were hormone and stress responses (BP), Ca^2+^ ion binding, and transporter activity (MF), and associated with macromolecular complexes and PS (CC), while small molecule metabolic processes (BP), catalytic activity (MF), and associated with mitochondria (CC) were decreased (Supplemental Table S3). This involved a preferential decrease in proteins associated with antioxidative functions, redox regulation, and flavonoid synthesis, as listed in Supplemental Table S1. In response to the FL shift, only very few categories showed significant changes, with GO terms photosynthesis (BP) and associated to chloroplast thylakoids (CC) being increased, while signaling, small molecule biosynthetic processes (BP), transferase activity (MF), and associated to plasma membrane structures (CC) were decreased (Supplemental Table S4). This involved a preferential increase in proteins involved in CBC and CEF (including PGR5) and redox processes (including Trx *m*4 activating CEF), as listed in Supplemental Table S1.

With respect to proteins that showed shared changes in all light shifts, the most significantly changed categories were redox processes (BP), oxidoreductase activity (MF), and chloroplast stroma association (CC) (Supplemental Table S5). The list of DEPs that showed shared changes in all light conditions (HL, LL, and FL) is shown in Table 2 and includes proteins involved in CEF (PGR5 and several NDH complex proteins), phenylpropanoid synthesis and oxylipin signaling as a core set of light-responsive marker proteins. These core proteins have mainly redox functions (Supplemental Table S5) and are suggested to be involved in general acclimation responses to a shift in light intensity or variability. This underlines the importance of thiol-redox networks in light acclimation responses.

**Table 2.** Core set of marker proteins with significant and common protein changes during acclimation to FL, HL, and LL (relative to ML) is mainly linked to *oxidation–reduction processes* (GO:0055114) in the WT

Locus	Protein names	Location
AT1G76680	12-oxophytodienoate reductase 1	cytosol
AT3G24503	Aldehyde dehydrogenase family 2 member C4	cytosol
AT4G39330	Probable cinnamyl alcohol dehydrogenase 9	cytosol
AT1G17990	Putative 12-oxophytodienoate reductase-like protein 2A	cytosol
AT1G79440	Succinate-semialdehyde dehydrogenase	mitochondrion
AT2G45770	Cell division protein FtsY homolog	plastid
AT5G21430	NAD(P)H-quinone oxidoreductase subunit U	plastid
AT4G22890	PGR5-like protein 1A	plastid
AT1G06690	Uncharacterized oxidoreductase	plastid

Significances on protein level were tested with a repeated *t*-test following Benjamini–Hochberg (Benjamini and Hochberg 1995) correction (*P* < 0.05). Before that, GO term enrichments were evaluated with “agriGO” (FDR < 0.05) (Tian et al. 2017). Location (consensus) was determined with SUBA4 (Hooper et al. 2017). For details see Supplemental Table S5 in the supplement. FDR, false discovery rate.

Compared to WT, *ntrc* and *trxm1m2* mutants revealed the strongest impact on the number of DEPs (Supplemental Fig. S8), specifically during HL acclimation, yielding 227 and 51 DEPs, respectively (Supplemental Fig. S8C). In the *ntrc* mutant, the changes in the respective functional categories were opposite to WT, providing evidence that the large-scale reprograming of the proteome during HL acclimation observed in the WT is almost completely abolished in response to NTRC deficiency (Supplemental Table S6). Interestingly, most of the HL-responsive DEPs in the *trxm1m2* mutant (43 out of 51) overlapped with those of the *ntrc* mutant (Supplemental Fig. S8C). In this HL overlap of *trxm1m2* and *ntrc*, the most significant categories included stress/external stimulus responses, chloroplast/thylakoids, and cytosol/ribosomes (Supplemental Table S7). This encompassed several proteins involved in oxidation–reduction processes, such as Trx *h*5, chloroplast Prx2E, chloroplast superoxide dismutase, glutathione S-transferase F2, succinate CoA ligase, and cinnamoyl-CoA reductase, as well as ribosomal subunit proteins (Supplemental Table S8), indicating that both mutants share common responses at the proteome level during HL acclimation. In comparison to this, the *trxf1* mutant showed only a minor impact on DEPs in ML, HL, and LL, while in FL, 29 DEPs were found in *trxf1* and only six DEPs in *trxm1m2* mutants, which were in both mutants mainly decreased (Supplemental Fig. S8B), indicating a role of Trx *f*1 in acclimation of the proteome in FL.

*In summary,* light-intensity dependent changes in the global proteome fall into two different clusters with functions related to translation and stress responses, revealing large-scale decreases and increases in protein abundance during HL acclimation, respectively, which are strongly attenuated in both *ntrc* and *trxm1m2* mutants.

### Correlation of protein level and growth light intensity is disturbed in *ntrc* and *trxm1m2* mutants

The results of the proteomic analysis described above suggest that in the WT, levels of cluster 1 and cluster 2 proteins are negatively and positively correlated to growth light intensity, respectively, while this correlation was impaired in *ntrc* and *trxm1m2* mutants (Fig. 3C). To investigate this interdependency in a more direct and quantitative manner, a regression analysis was performed, comparing protein levels and photon flux density in the different genotypes. The numbers of significantly changed regression coefficients for all proteins are shown in Fig. 3E. In WT, the abundance of approximately 500 or 600 proteins positively or negatively correlated with light intensity, respectively. Compared to this, the number of total proteins showing significant correlations to light intensity was dramatically decreased in *ntrc* (100 positive/100 negative) and *trxm1m2* mutants (100 positive/50 negative), while the *trxf1* mutant resembled WT. This confirms that the re-engineering of the proteome in response to increased growth-light intensities is strongly attenuated in *ntrc* and *trxm1m2* mutants.

Since increased light intensity showed strong effects on photosynthetic performance (Figs. 1B and 2 and Supplemental Fig. S1B), we analyzed regression coefficients specifically for photosynthetic proteins encoded in the chloroplast genome (Fig. 3F). In the WT, four proteins were positively correlated with light intensity, while 28 proteins were negatively correlated in a significant manner. These numbers were severely decreased in *ntrc* (3 positive/7 negative) and *trxm1m2* mutants (0 positive/4 negative), while the *trxf1* mutant behaved like WT. In Supplemental Fig. S9, the regression coefficients of these individual photosynthetic proteins are displayed on a map showing different categories of light reactions. As already indicated in Fig. 3F, the levels of most of the photosynthetic proteins were negatively correlated with light intensity, which involved almost all proteins of the light-harvesting complexes, PS I and II, cytochrome *b*_6_*f* complex, photosynthetic electron transport, and NADH dehydrogenase, while proteins of *f*-type ATPase showed mainly positive correlations (Supplemental Fig. S9). This confirms previous studies showing similar association patterns of photosynthetic proteins with light intensity in the WT (Flannery et al. 2021; von Bismarck et al. 2023). In *ntrc* and *trxm1m2* mutants, these correlations were weaker, less significant, or opposite to WT, specifically with respect to proteins of the 2 PS, photosynthetic electron transport, cytochrome b6f complex, NADH dehydrogenase, and *f*-type ATPase (Supplemental Fig. S9). This indicates that deficiencies in NTRC or Trxs *m*1/*m*2 disturb the regulation of photosynthetic protein levels in response to increased growth-light intensity.

### Acclimation in HL involves large dynamics in the global metabolome that are strongly restricted in the *ntrc* and partly in the *trxm1m2* mutant

As outlined above, there was a large re-programming of the proteome, specifically in response to HL acclimation, which was strongly attenuated in response to NTRC or Trxs *m*1/*m*2 deficiencies. To investigate whether this was paralleled by global changes in metabolite levels, gas chromatography (GC)-mass spectrometry (MS) was used to analyze metabolite profiles in WT, *trxf1*, *trxm1m2,* and *ntrc* mutants 7 days after the shifts from ML to LL, HL, or FL. Rosettes were sampled 6 h into the photoperiod. Since metabolite levels can alter within seconds to minutes after a change in light intensity, rosettes were rapidly frozen in liquid nitrogen during illumination with the respective light intensity before extraction and analysis. With respect to FL, rosettes were harvested in the LL (4 min) and HL phases (1 min) of FL separately.

**Figure 4.**
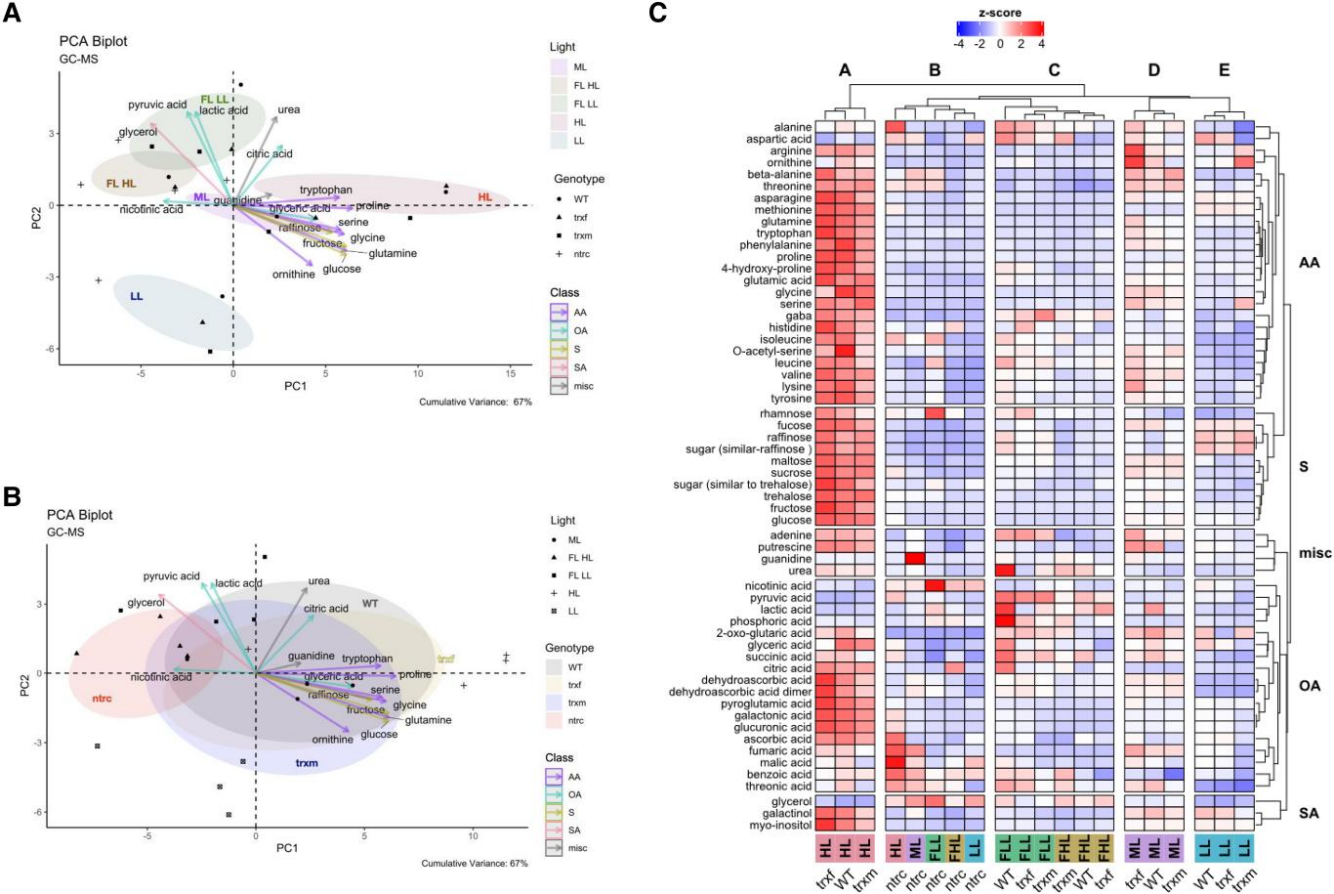
Cluster analysis of light-dependent and genotypic changes of the global metabolome. WT, *trxf1* (*trxf*), *trxm1m2* (*trxm*), and *ntrc* mutants were shifted for 7 days into different acclimation conditions (ML, LL, HL, or FL, for details see legend of Fig. 2), before rosettes were sampled 6 h into the photoperiod to analyze changes in the GC-MS based global metabolome using principle component analysis (PCA) and cluster analysis. **A, B)** PCA of global metabolites, according to light (A) or genotype (B). Only the most important metabolites were plotted. The cumulative variance of PC1 and PC2 is 67%. The clustering was done with the package “factoextra” and “FactoMineR” in R. **C)** Heat map of standardized global metabolite levels, showing z-scored changes in metabolite levels, with blue color indicating down-regulation, while red color indicating up-regulation with respect to the mean of all samples (generated with R package “ComplexHeatmap”). AA, amino acid; S, sugar; misc, miscellaneous; OA, organic acid; SA, sugar alcohol; FLL, LL phase of FL; FHL, HL phase of FL. Raw data and statistics, see Supplemental Table S9 and Fig. S10, respectively.

For a first overview, metabolite data (Supplemental Table S9) were used to create a PCA to provide information about the relationships between different light conditions and genotypes and the most important contributing metabolites. With respect to light conditions, principle component (PC) biplot analysis of the metabolome data shows that the metabolite profiles clearly clustered differently in response to seven-day shifts from ML to HL, LL, or FL (Fig. 4A). Interestingly, there was also an additional separation between the metabolite profiles of the LL and HL phases of FL, indicating that long-term and short-term changes in light intensities lead to different shifts in metabolite profiles. This was captured in both PC1 and PC2, with separation in PC1 being mainly driven by tryptophan, glyceric acid, proline, serine, glycine, glucose, fructose, and raffinose (higher in HL), while separation in PC2 being driven by citric acid, pyruvic acid, lactic acid, urea and glycerol (higher in the LL phases of FL). With respect to different genotypes, the PCA in Fig. 4B shows that the metabolite profiles of *trxf1* and *trxm1m2* mutants were similar to WT (with *trxm1m2* being a bit more different), while metabolites of the *ntrc* mutant fell in a clearly different cluster. This was captured mainly in PC1, with separation being driven by tryptophan, glyceric acid, proline, serine, glycine, glucose, fructose and raffinose (lower in *ntrc*), and nicotinic acid and glycerol (higher in *ntrc*). This shows that a shift in the irradiance has strong effects on metabolite profiles, while genotypic effects are relatively small, except for *ntrc*.

To get a more detailed overview of the global changes in metabolite profiles, we used cluster analysis of the z-scored data. The results are summarized in the heatmap of Fig. 4C displaying five different metabolite clusters, revealing light-dependent (clusters A, C, D, E) and genotypic changes in metabolite profiles (cluster B). In the WT, clusters A, C, D, and E represent different metabolite profiles specific for HL, FL, ML, and LL, respectively, with cluster C falling into two subclusters representing metabolite profiles in the LL and HL phases of FL. In constant light, there was a general increase in metabolite levels with increasing light intensity, with most metabolites being low in LL (cluster E), at an intermediate level in ML (cluster D) and high in HL (cluster A), specifically with respect to almost all amino acids and sugars and several organic acids, except aspartic acid, nicotinic acid, pyruvic acid, lactic acid, phosphoric acid and glycerol which remained low. In FL, the metabolite profiles were different from those found in constant light conditions, with most metabolites except a few organic acids being low in the HL phases of FL, while subsets of amino acids (e.g. alanine, aspartic acid, and GABA), organic acids (e.g. pyruvic acid, lactic acid, phosphoric acid, 2-oxo glutaric acid, glyceric acid, succinic acid and citric acid) and precursors of nucleotides (adenine and urea) were high in the LL phases of FL. With respect to different genotypes, metabolite profiles of *trxf1* and *trxm1m2* mutants were similar to WT, while the *ntrc* mutant clustered clearly differently from all other genotypes with respect to all light conditions (cluster B). Specifically, HL-induced changes in metabolite levels were strongly suppressed in the *ntrc* mutant, with almost all metabolites (specifically sugars, amino acids, and most organic acids) remaining low, except for a few organic acids (e.g. ascorbic acid, fumaric acid, malic acid, and benzoic acid) and alanine being increased in *ntrc*, but not in the other genotypes. Interestingly, a more detailed inspection of Fig. 4C shows that HL induction of metabolite levels (specifically with respect to amino acids) was also slightly decreased in the *trxm1m2* mutant when compared to WT or *trxf1*.

Quantitative dynamics of global metabolite levels are summarized in Supplemental Fig. S10 and visualized in a genotype-dependent manner in the violin plot of Supplemental Fig. S11. Supplemental Fig. S10 shows that genotypic variations in individual metabolite levels, specifically most sugars (specifically sucrose, glucose, fructose, maltose, and trehalose), amino acids (glutamic acid, glutamine, glycine, methionine, phenylalanine, proline, tryptophan, and tyrosine), starch and (dehydro)ascorbate revealed the largest dynamics in HL acclimated plants, while these dynamics were clearly dampened when light intensity was decreased during the acclimation phase. In Supplemental Fig. S11, light-dependent variations in the levels of individual metabolites are shown in different genotypes. The levels of almost all metabolites involved in sugar, AA, and organic acid metabolism (except guanidine, malic acid, and nicotinic acid) revealed large light-dependent dynamics in WT, *trxf1* and *trxm1m2* mutants, while those measured in the *ntrc* mutant were severely restricted, with values being mainly distributed to lower levels (except benzoic acid, glycerol, malic acid and nicotinic acid showing higher dynamics and levels in the *ntrc* mutant compared to the other genotypes). For selected metabolites (i.e. fructose, fucose, and asparagine), the *trxm1m2* mutant showed restricted light-dependent dynamics, although not as strong as in *ntrc*. This indicates that NTRC and partly Trxs *m*1/*m*2 have a strong influence on the dynamics of sugars, starch, and amino acids in response to a shift in light intensity.

*In summary*, a long-term increase in irradiance is associated with a marked rise in the levels of photosynthetic end-products such as sugars, starch, and amino acids, which are strongly restricted in response to NTRC or partly Trxs m1/*m*2 deficiencies, indicating these thiol-redox regulators to play an important role in metabolic acclimation to increased light intensity.

### Acclimation to increasing light intensities involves a coordinated increase in the intermediates of the Calvin–Benson cycle and related pathways, which is disturbed in *ntrc* and *trxm1m2* mutants

**Figure 5.**
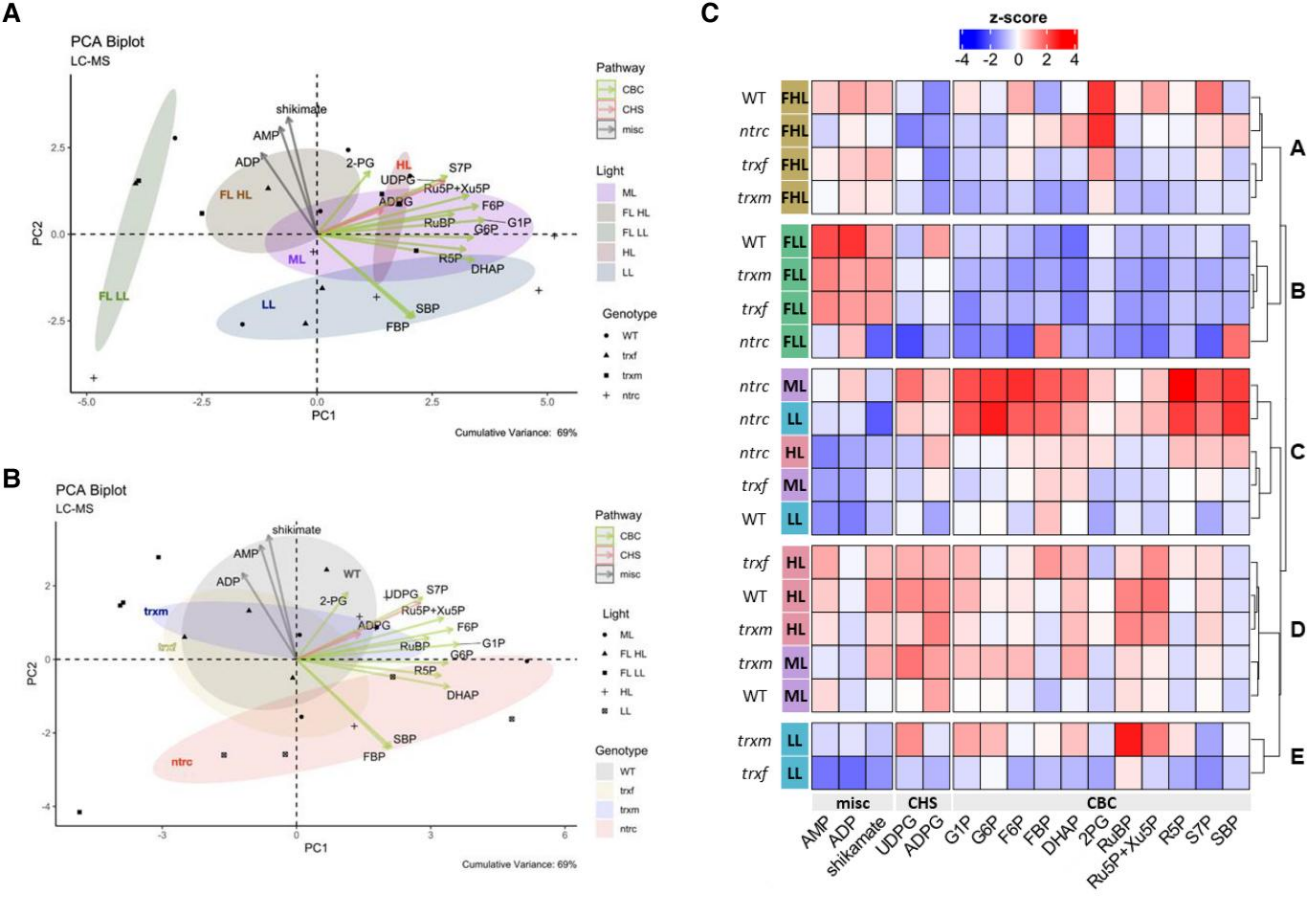
Cluster analysis of light-dependent and genotypic changes of the Calvin–Benson-cycle related metabolome. WT, *trxf1* (*trxf*), *trxm1m2* (*trxm*), and *ntrc* mutants were shifted for 7 days into different acclimation conditions (ML, LL, HL, or FL, for details see legend of Fig. 2), before rosettes were sampled 6 h into the photoperiod to analyze changes in the LC-MS/MS based Calvin–Benson-cycle (CBC) related metabolome using principle component analysis (PCA) and cluster analysis. **A, B)** PCA of CBC-related metabolites, according to light (A) or genotype (B). Only the most important metabolites were plotted. The cumulative variance of PC1 and PC2 is 69%. The clustering was done with the package “factoextra” and “FactoMineR” in R. **C)** Heat map of standardized CBC-related metabolite levels, showing z-scored changes in mean values of metabolite levels, with blue color indicating down-regulation, while red color indicating up-regulation with respect to the mean of all samples (generated with R package “ComplexHeatmap”). CBC, Calvin–Benson-cycle; CHS, carbohydrate synthesis; misc, miscellaneous; FLL, LL phase of FL; FHL, HL phase of FL. Raw data and statistics see Supplemental Table S10 and Fig. S12, respectively.

Since long-term alterations in light intensities were found to induce changes in the photosynthetic proteome and photosynthetic end-products, as outlined above, we were interested in analyzing the levels of intermediates of photosynthetic metabolism under these conditions in more detail. To do this, we used a liquid chromatography (LC)-MS/MS method to analyze a nearly full set of the intermediates of the CBC and selected related metabolites (Supplemental Table S10), involved in photorespiration (2-phosphoglycolate [2PG]), starch synthesis [ADPG], sucrose synthesis (UDP-glucose [UDPG]) and erythrose-4-P (E4P) metabolism (shikimate), or serving as nucleotide cofactors (ADP and AMP). PCA of the metabolite data showed that photosynthetic metabolite profiles clustered differently in response to the shifts from ML to HL, LL, or FL (Fig. 5A). With respect to long-term dynamics in constant light intensities, the photosynthetic metabolite profiles in LL, ML, and HL were clustering separately. This separation was mainly captured in PC1, being driven by sedoheptulose 1,7-bisphosphate (SBP) and fructose 1,6-bisphosphate (FBP) (higher in LL), and ADP, AMP, and shikimate (lower in LL). Interestingly, metabolite profiles in FL were clustered separately from those in constant light intensities. When the different FL phases were compared, there was a specifically clear separation between the metabolite profiles of the LL and HL phases of FL, indicating short-term dynamics in metabolite levels within time frames of 1 to 5 min, which were largely captured in PC2, with separation in PC2 being mainly driven by glucose 6-phosphate (G6P), fructose 6-phosphate (F6P), glucose 1-phosphate (G1P), ribulose 1,5-bisphosphate (RuBP), ribulose 5-phosphate (Ru5P), sedoheptulose 7-phosphate (S7P), and ADPG (mainly lower in the LL phase of FL). With respect to different genotypes, the PCA in Fig. 5B shows that the metabolite profiles of *trxf1* were similar to WT, although *trxm1m2* revealed lower dynamics of metabolite changes than WT, while the *ntrc* mutant clustered clearly differently. This was captured mainly in PC1, with separation being driven by SBP, FBP, and dihydroxyacetone-phosphate (DHAP) (higher in *ntrc*), and shikimate, ADP and AMP (lower in *ntrc*). This shows that both short-term and long-term shifts in irradiance exert strong effects on the levels of intermediates of the CBC and related pathways, while genotypic effects are minor with respect to *trxf1,* relatively small in *trxm1m2*, but large in *ntrc* mutants.

**Figure 6.**
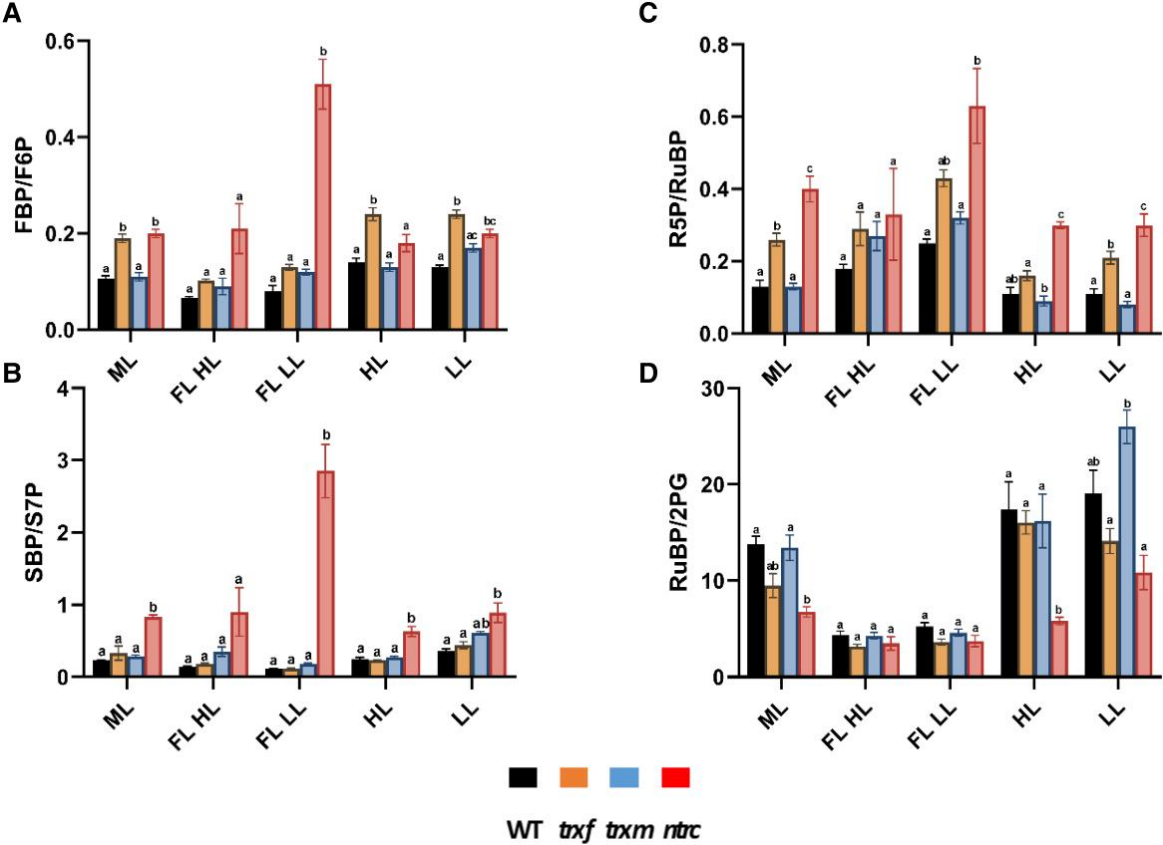
Changes in the ratios of Calvin–Benson-cycle related metabolite levels in WT, *trxf1*, *trxm1m2,* and *ntrc* mutants after acclimation to different light conditions. WT (black), *trxf1* (*trxf*, yellow), *trxm1m2* (*trxm*, blue), and *ntrc* (*ntrc*, red) mutants were shifted for 7 days into different acclimation conditions (ML, LL, HL, or FL, for details see legend of Fig. 2), before rosettes were sampled 6 h into the photoperiod to analyze changes in the levels of LC-MS/MS based Calvin–Benson-cycle related metabolites to calculate metabolite ratios. **A)** FBP/F6P. **B)** SBP/S7P. **C)** R5P/RuBP. **D)** RuBP/2PG. Results are the mean ± SE, *n* = 3 to 5 biological replicates. Significance levels within one condition were evaluated by using a one-way ANOVA with a post hoc Tukey test (*P* < 0.05) and were labeled with different letters. FL LL, LL phase of FL; FL HL, HL phase of FL. Raw data see Supplemental Table S10.

To get an overview of the dynamics of the metabolic intermediates of the CBC and selected metabolites in related pathways, we used cluster analysis of the z-scored data. The results are summarized in the heatmap of Fig. 5C, showing five different metabolite clusters, revealing two clearly separated clusters with respect to the HL (cluster A) and LL (cluster B) phases of FL, while the different constant light conditions LL, HL, and ML were distributed between clusters C-E. In the WT, clusters A, B, and C represented different metabolite profiles specific for FL-HL, FL-LL, and LL, respectively, while the metabolite profiles for HL and ML were assigned to different subclusters within cluster D. As shown in the heatmap of Fig. 5C and in the more quantitative boxplot of Supplemental Fig. S12, a long-term increase in light intensity in the WT was found to be attributed to a general increase in the levels most of the intermediates of the CBC, with the exception of FBP and SBP staying at low levels due to increased light activation of FBPase and SBPase, confirming previous studies (Borghi et al. 2019). This was accompanied by a rise in the key intermediates of photorespiration (2PG), starch synthesis (ADPG), sucrose synthesis (UDPG), E4P metabolism (shikimate), and nucleotide cofactors (ADP and AMP), indicative of a stimulation of the CBC to decrease acceptor side limitation of ATP synthase and PSI and to increase end-product synthesis (compare Supplemental Figs. S10 and S12). Compared to the differences in metabolite levels between WT plants acclimated to constant HL or LL, short-term metabolic dynamics between HL and LL phases of FL showed similar characteristics with respect to the intermediates of the CBC and photorespiration (2PG), which were generally increased in HL compared to LL periods, while FBP and SBP remained unaltered (Fig. 5C and Supplemental Fig. S12). When different genotypes were compared during acclimation to constant light intensities, the CBC metabolite profiles of the *ntrc* mutant clustered differently to WT, specifically with respect to ML and LL, where all intermediates of the CBC, except RuBP, were much higher in the *ntrc* mutant, compared to the other genotypes (Fig. 5C and Supplemental Fig. S12). In constant LL and ML, NTRC deficiency led to specifically strong increases in SBP (6-fold) and FBP (3-fold) levels (Supplemental Fig. S12) and SBP/S7P (4-fold) and FBP/F6P (2-fold) ratios (Fig. 6, A and B), which are most likely attributable to incomplete photoreduction of FBPase and SBPase, respectively (for control G6P/G1P and G1P/ADPG ratios showed only minor changes, except of an increase of the latter in *ntrc* in ML, see Supplemental Fig. S13). These metabolic changes in the *ntrc* mutant were clearly attenuated under HL conditions, yielding smaller increases in CBC intermediates than in LL and ML or even decreased levels with respect to RuBP and G1P. While the CBC metabolite profiles of the *trxf1* mutant were largely similar to WT, those of the *trxm1m2* mutant revealed a pattern more similar to *ntrc*, with the levels of CBC intermediates being mainly higher under LL and ML rather than HL conditions, although this effect was less marked than in the *ntrc* mutant. Interestingly, the effects of Trx *f*1-deficiency on FBP and RuBP levels (Fig. 5C and Supplemental Fig. S12) and FBP/F6P and R5P/RuBP ratios (Fig. 6, A and C), suggesting a role of Trx *f*1 in regulating FBPase, see (Thormählen et al. 2015), and phosphoribulokinase (PRK) in vivo, were found in all constant light conditions (ML, LL, and HL). This indicates that Trx *f*1-dependency of CBC enzymes does not vary with light intensity.

Genotypic alterations of overall levels of CBC and related metabolites were also very high in FL. Specifically, NTRC deficiency led to a strong increase in the levels of SBP and FBP, which were more marked in the LL phase (4 to 6 fold) than in the HL phase of FL (3 to 4 fold). Conversely, the levels of S7P, F6P, and RuBP decreased (Supplemental Fig. S12), while the SBP/S7P (30-fold), FBP/F6P (6-fold), and R5P/RuBP ratios (3-fold) severely increased (Fig. 6, A to C), indicating an incomplete photoreduction of the CBC key enzymes SBPase, FBPase, and PRK in response to NTRC deficiency, which was specifically marked in the LL phases of FL. This is in line with the idea that NTRC upholds the reduction of enzymes at lower light intensities, while Fdx-Trxs act at higher light intensities (Carrillo et al. 2016; Thormählen et al. 2017). In addition, NTRC deficiency led to a decrease in the levels of shikimate, specifically in the LL phases of FL (Supplemental Fig. S12), indicating a possible role of NTRC in E4P metabolism. These data indicate that NTRC deficiency leads to a strong imbalance in CBC intermediates that will impair photosynthesis during acclimation to FL intensities.

*In summary*, while NTRC and Trxs *m*1/*m*2 are important to allow a coordinated increase in CBC intermediates during acclimation to light intensity, there is a specific role of NTRC to balance CBC metabolite levels to stabilize photosynthetic activity during acclimation to light variability.

### Correlation of metabolite levels and growth light intensity is de-regulated in *ntrc* and *trxm1m2* mutants

**Figure 7.**
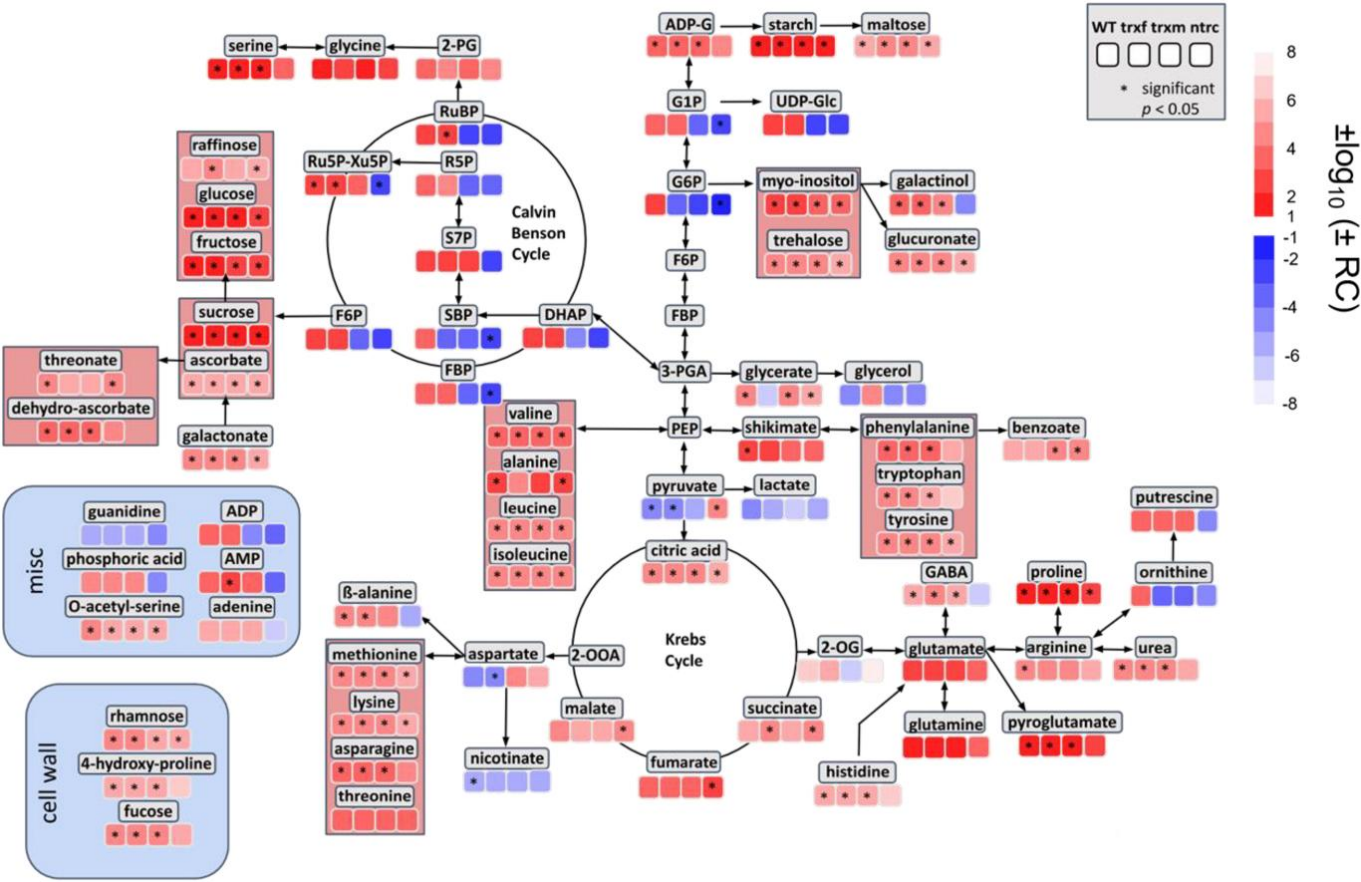
Quantitative correlation of metabolite levels and light intensity in WT, *trxf1*, *trxm1m2*, and *ntrc* mutants during acclimation. Linear regression analysis of GC-MS (Supplemental Table S9) and LC-MS/MS-based metabolites (Supplemental Table S10) was performed using acclimation-light intensity (photon flux density in µE) as independent variable. The heat map shows log10-transformed regression coefficients, where transformation of positive coefficients was multiplied by −1 while negative coefficients were multiplied by −1 prior to transformation (values closer to 1 or −1, respectively, indicate a stronger correlation with light intensity). Significance levels (*P* < 0.05) are labeled with asterisks. RC, regression coefficient; WT, wild type; trxf, *trxf1;* trxm, *trxm1m2;* ntrc, *ntrc*.

Our results of GC-MS and LC-MS/MS analyses described above indicate that there is a positive correlation between metabolite level and light intensity in the WT. To investigate this relationship in a more direct and quantitative manner, a regression analysis was performed comparing metabolite levels and photon flux density. The regression coefficients for individual metabolites in different metabolic pathways are shown in Fig. 7. In the WT, there is a positive correlation between metabolite levels and light intensity for almost all intermediates of the CBC and related pathways, even in extra-plastidial and respiratory metabolism, which is significant for most of the metabolites. Interestingly, only very few metabolites, such as pyruvate, aspartate, or nicotinate showed a negative correlation with increasing light intensity. A similar correlation pattern is also seen in the redox mutants, indicating that the correlation between metabolites and irradiance is, in general, still robust. However, in the *ntrc* and *trxm1m2* mutants, the positive correlation between metabolites and light intensity is clearly disturbed with respect to the intermediates of the CBC, hexose-phosphate metabolism, and ADP, showing negative regression coefficients. This finding suggests a de-regulation of these pathways in *ntrc* and *trxm1m2* mutants during acclimation in increasing light intensities. In contrast to this, the *trxf1* mutant was similar to WT. These results indicate that NTRC and, to a lesser extent, Trxs *m*1/*m*2 are both important to regulate the CBC and related pathways in response to acclimation to light intensity.

## Discussion

**Figure 8.**
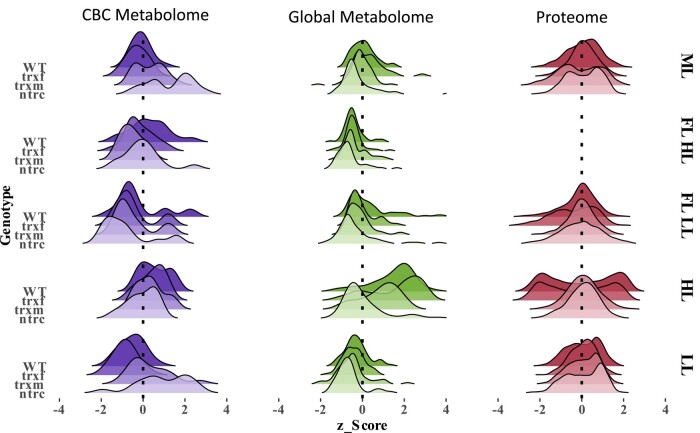
Overview of metabolic and proteomic changes in WT, *trxf1*, *trxm1m2,* and *ntrc* mutants acclimated to different light conditions. In each category all variables were pooled and standardized across all light conditions (ML, FL HL, FL LL, HL, and LL, for details, see legend to Fig. 2) to form the ridgeline shaped curves, expressing the density of changes within one sample compared to other samples. CBC, Calvin–Benson-cycle; FL LL, LL phase of FL; FL HL, HL phase of FL; WT, wild type; trxf, *trxf1;* trxm, *trxm1m2;* ntrc, *ntrc*. Raw data see Supplemental Tables S1, S9, and S10.

In natural dynamic light environments, photosynthesis has to manage strong fluctuations in light availability on different time scales, leading to long-term acclimation as well as short-term responses of plants. In this study, we investigated how light environmental factors such as light intensity or variability affect long-term acclimation responses of *Arabidopsis* plants in different light environments at the levels of the proteome and metabolome and how this influences photosynthetic dynamics and plant growth. Results show that in the WT, an increase in light intensity during HL acclimation leads to large quantitative changes in the proteome and metabolome (Fig. 8), which are accompanied by increased dynamics of NPQ and PSII quantum efficiency during short-term light fluctuations (Fig. 2) as well as by increased plant growth rates (Supplemental Fig. S1A). Compared to this, changes in the proteome and metabolome are less marked in response to FL (Fig. 8), while photosynthetic dynamics and plant growth are decreased under these conditions (Figs. 1B and 2, Supplemental Fig. S1A). This supports that light intensity is a stronger driver for acclimation than variability.

Using *Arabidopsis* mutants lacking parts of the Fdx-Trx or NADPH-NTRC systems, we investigated the importance of the chloroplast thiol-redox network in this context. Our results show that NTRC and Trxs *m*1/*m*2 are both indispensable for the large-scale re-engineering of the proteome and metabolome during HL acclimation (Fig. 8), while NTRC is also crucial to ensure improved photosynthetic dynamics and increased plant growth under these conditions (Figs. 1, A and B and 2, Supplemental Fig. S1, A and B). In addition to this, NTRC is important to optimize CBC activity, photosynthetic dynamics and plant growth during acclimation in FL, while Trx *f*1 plays only minor roles in HL or FL acclimation responses despite a slight involvement in the FL response of the proteome. This shows that different parts of the chloroplast thiol-redox network exert different roles in light acclimation responses.

### Light intensity is a stronger driver for acclimation than variability, leading to large-scale remodeling of the proteome and metabolome

As summarized in the ridgeline plots of Fig. 8, our results show that HL, but not FL, acclimation is associated with large quantitative changes in global proteome and metabolome, indicating light intensity to be a stronger driver for acclimation than variability. During HL acclimation, proteins involved in photosynthetic light reactions (Supplemental Figs. S2H and S9) were mainly decreased, while those of *f-*type ATPase, CBC, and carbon metabolism (including respiration) were increased (Supplemental Table S2 and Fig. S9), which is in line with the increase in metabolic intermediates of the CBC and related pathways (Fig. 5 and Supplemental Fig. S12) and their products, i.e. sugars, amino acids, organic acids, and starch (Fig. 4 and Supplemental Fig. S10), leading to an improved capacity and efficiency of photosynthesis (Supplemental Fig. S1B, Figs. 1B and 2). Intriguingly, regression analyses show that almost all metabolite levels across diverse pathways were positively correlated with light intensity (Fig. 7). This confirms and extends previous studies on HL acclimation, revealing an increased abundance of proteins involved in photosynthetic electron transport and carbon metabolism (Miller et al. 2017) and increased levels of photosynthetic end-products (Garcia-Molina et al. 2020) when light intensity is increased. In addition to these changes related directly to photosynthesis, proteins involved in antioxidative functions, stress responses, CEF, redox regulation and flavonoid synthesis were found to be increased (Supplemental Figs. S2, E and F, S6A and Table S2), which was accompanied by increased metabolic levels of ascorbate, shikimate and benzoic acid (Fig. 4 and Supplemental Fig. S10), leading to increased photoprotective capacities (Fig. 1B). Interestingly, marker proteins important for NPQ formation, such as PsbS (Supplemental Fig. S9) or enzymes of zeaxanthin metabolism, such as carotene hydroxylase and violaxanthin de-epoxidase (Supplemental Fig. S6A and Table S1) were decreased in response to HL, which is in line with the down-regulation of NPQ during short-term photosynthetic dynamics in HL-acclimated plants (Supplemental Fig. S1B and Fig. 2).

HL acclimation also led to a previously unknown large-scale and highly significant decrease in proteins involved in translation, ribosomal structures, and ribosome biogenesis, mainly in the cytosol but also in the plastid (Supplemental Fig. S2G and Fig. 3C; Table 1 and Supplemental Tables S2 and S11). This involved a significant decrease in a large number of ribosomal proteins in the cytosol (63 proteins) and plastid (7 proteins) (Supplemental Table S11). A role for plastid and cytosolic ribosomal proteins in abiotic stress responses has been proposed previously in various plant species (Kim et al. 2004; Wang et al. 2013a). Translational regulation in both subcellular compartments has been found to be specifically important during cold stress (Guo et al. 2002; Rogalski et al. 2008; Liu et al. 2010; Zhang et al. 2016; Wang et al. 2017), but there were also reports indicating that translation factors in the cytosol are important for tolerance to HL (Lokdarshi et al. 2020) and heat (Zhang et al. 2017; Li et al. 2018). Recent transcriptome studies are in line with this notion (Garcia-Molina et al. 2020), showing transcripts for ribosomal proteins to be down-regulated at the end of the HL acclimation phase (after 2 to 4 days), which agrees with our proteome data (Supplemental Fig. S2G and Fig. 3C: Table 1 and Supplemental Table S2). A suppression of translation and protein synthesis may decrease protein turnover and save ATP, which could be beneficial to promote increased growth rates (Ishihara et al. 2017). Interestingly, oxidative stress treatments were found to negatively affect the number of ribosomes in plants (Salih et al. 2020), while ROS produced in the chloroplast under HL conditions was found to affect the status of the cytosolic protein synthesis apparatus to suppress cytosolic translation (Lokdarshi et al. 2020). In the present study, HL acclimation was accompanied by an increase in several parameters that are indicative of oxidative stress, such as a decrease in the Fv/Fm ratio (Fig. 1B), increases in ascorbate levels (Fig. 4) and proteins involved in oxidative stress responses (Supplemental Fig. S2F and Table S2); thus, the re-engineering of the translational machinery in cytosol and plastids may have HL-induced ROS or redox signals as possible triggers. More studies will be necessary to explore whether this is associated with a decrease in ribosomal numbers or a change in ribosomal composition/activity and to analyze the underlying mechanisms and signals involved.

### NTRC stabilizes the CBC in FL and underpins long-term acclimation to light intensity at the proteome and metabolome level

**Figure 9.**
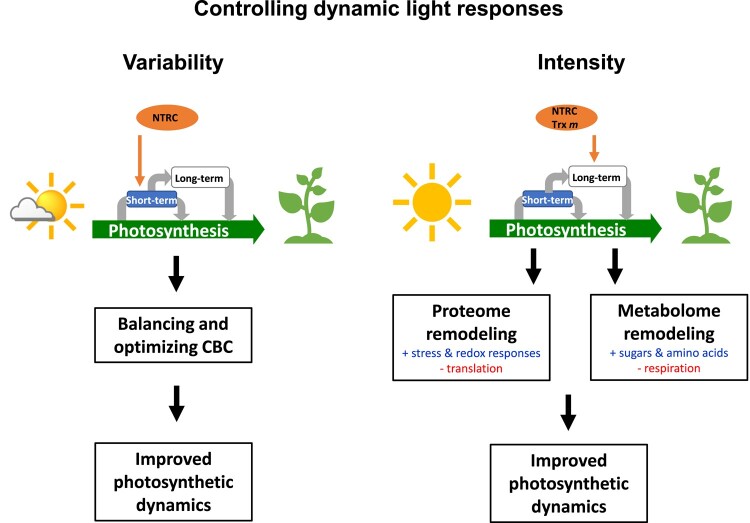
Schematic model of the role of NTRC and Trxs *m*1/*m*2 in controlling dynamic light responses. In natural dynamic light environments, photosynthesis has to manage strong fluctuations in light availability on different time-scales, leading to long-term acclimation as well as short-term-responses of plants. NTRC plays a major role in controlling short-term responses of photosynthesis during rapid light fluctuations being indispensable to optimize photosynthesis and plant growth during acclimation to light variability. In this context, NTRC is important to balance the CBC to allow improved dynamics of photosynthetic light reactions. In addition to this, both, NTRC and Trxs *m*1/*m*2 support the re-engineering of the proteome and metabolome during long-term acclimation to light intensity. During prolonged growth in elevated light, these redox regulators trigger intra- and interorganellar signals to control cytosolic and chloroplast translation as well as stress and redox responses to improve photosynthetic dynamics, end-product synthesis, and plant growth. Trx *m*, Trxs *m*1/*m*2.

NTRC is a chloroplast thiol-redox system that receives its reducing potential from NADPH and provides electrons to target proteins via its own tethered Trx domain (Serrato et al. 2004). While NTRC is not able to control the reduction state of CBC enzymes directly (Ojeda et al. 2017), it uses NADPH to reduce 2-Cys-peroxiredoxins (2Cys-Prx), which indirectly modulates the reduction state of a whole set of Trx-regulated target enzymes in response to dark-light transitions in the chloroplast (Perez-Ruiz et al. 2017). Moreover, NTRC has been found to be indispensable for photosynthetic acclimation in FL, although the underlying mechanisms remained unclear. Here, we show that NTRC deficiency impaired photosynthetic dynamics and plant growth in FL (Figs. 1 and 2), although this was not accompanied by marked changes in the proteome and global metabolome (Fig. 8). However, there were hyper-strong effects on SBP/S7P (30 fold), FBP/F6P (6-fold), and R5P/RuBP (3-fold) ratios, specifically in the LL phases of FL (Fig. 6), indicating NTRC deficiency to severely attenuate the dynamics of redox-regulation of SBPase, FBPase, and PRK, which are key enzymes of the CBC. This suggests that NTRC has an important role in stabilizing CBC activity to optimize photosynthetic dynamics and plant growth during acclimation to light variability (Fig. 9).

Our results show that in addition to this, NTRC deficiency strongly attenuates HL acclimation at the level of photosynthesis (Figs. 1B and 2) and plant growth (Fig. 1A and Supplemental Fig. S1A). As summarized in the ridgeline plots of Fig. 8, this is accompanied by an almost complete suppression of the largescale remodeling of the proteome (Fig. 3 and Supplemental Fig. S3) and metabolome (Figs. 4 and 5) characteristic for HL acclimation. In HL, NTRC deficiency prevents the quantitative shifts in the proteome to induce proteins involved in stress and redox responses and to repress those involved in protein synthesis and translation, as well as the increase in global and CBC metabolite levels (Figs. 3, C and D, 4, 5 and 8). The data from analyzing the correlation of protein or metabolite levels with light intensity suggest that the perturbation in HL acclimation is part of a general defect in the light-dependent plant acclimation response of the *ntrc* mutant (Figs. 3, E and F and 7). In this context, NTRC deficiency prevented the HL-responsive decrease of a large set of proteins involved in translation, ribosome biogenesis, and ribosome structure located in the cytosol, but also in the chloroplast (Supplemental Fig. S3 and Fig. 3D: Supplemental Tables S6 and S12), indicating a key role of NTRC in triggering light acclimation at the level of cytosolic and chloroplast translation to control cellular protein homeostasis. This is in line with previous studies, providing evidence that many components of the translational machinery in both subcellular compartments are subject to thiol-disulfide modulation (Trebitsh and Danon 2001; Moore et al. 2016). Additionally, in the chloroplast, many translational components were identified as potential Trx targets (Montrichard et al. 2009), and most of the recently identified NTRC-interacting proteins were classified to be involved in chloroplast protein synthesis, including 50S and 30S ribosomal proteins and translation initiation factors (González et al. 2019). Moreover, NTRC was proposed to be involved in thiol-disulfide regulation of a chloroplast translation factor to modulate light regulation of photosynthetic gene expression in *Chlamydomonas* (Schwarz et al. 2012). While this provides a possible mechanism for NTRC to modulate chloroplast translation via intra-organellar thiol-disulfide interactions, it remains unclear how NTRC is able to affect translational components beyond the chloroplast boundaries.

In addition to the strong effects on translational components, NTRC deficiency prevented the HL acclimation of the proteome at the level of photosynthetic light reactions, CEF, stromal metabolism, zeaxanthin synthesis, antioxidative functions, and redox regulation (Supplemental Figs. S3, E to H, S6B and S9, Fig. 3D). This was associated with a corresponding repression of HL-induced changes in the metabolome at the level of the synthesis of photosynthetic end products (i.e. sugars, amino acids, and starch: Fig. 4 and Supplemental Fig. S10) and intermediates of the CBC (Fig. 5 and Supplemental Fig. S12). Regression coefficients showed an impaired correlation between the levels of CBC intermediates and light intensity in response to NTRC deficiency, indicating a de-regulation of the CBC in the *ntrc* mutant. These findings most likely explain why NTRC deficiency prevented (i) long-term recovery of Fv/Fm, (ii) improved photosynthetic dynamics, and (iii) increased plant growth during HL acclimation.

As summarized in the model in Fig. 9, NTRC plays a major role in short-term acclimation to light variability to balance the CBC during rapid light fluctuations, as well as in long-term acclimation to light intensity to support the remodeling of the proteome and metabolome.

### Trxs *m*1 and *m*2 are important to support the remodeling of the proteome and metabolome during long-term acclimation to elevated light intensity

Being part of the light-dependent Fdx-Trx system, Trxs *m*1 and *m*2 comprise more than 50% of all chloroplast Trxs (Okegawa and Motohashi 2015). Both *m*-type Trx isoforms were found to affect photosynthetic performance in FL environments, with combined deficiencies in Trxs *m*1 and *m*2 in Arabidopsis *trxm1m2* mutants leading to lower photosynthetic efficiency in the HL periods but surprisingly higher photosynthetic efficiency in the LL phases of FL (Thormählen et al. 2017). The results of the present paper show that this feature refers to a short-term response that is independent of the acclimation conditions (Fig. 2) and does not lead to any growth effects (Fig. 1A). Focusing on FL conditions, Trxs *m*1/*m*2 deficiencies were not associated to substantial changes in the proteome and metabolome and in the ratios of CBC intermediates, suggesting these types of Trxs to be less important in acclimation responses to light variability (Fig. 9). However, we found that the *trxm1m2* mutant is strongly attenuated in the remodeling of the proteome during HL acclimation in a similar manner as the *ntrc* mutant (Figs. 3D, 8, Supplemental Figs. S4, E to H and S6C). In response to Trxs *m*1/*m*2 deficiencies, correlation of protein levels and light intensity was severely attenuated or disturbed (Fig. 3, E and F), showing that Trxs *m*1/*m*2 are important to regulate global protein levels (Fig. 3E) as well as protein levels of photosynthetic complexes during HL acclimation (Fig. 3F). This is in line with a previously published role of *m*-type Trxs in the accumulation of thylakoid protein complexes and biogenesis of the photosynthetic apparatus (Wang et al. 2013b). In agreement with this, combined deficiencies in Trxs *m*1 and *m*2 delayed the recovery of the maximum quantum efficiency of PSII (Fig. 1B) and disturbed the correlation of light intensity and CBC metabolite levels (Fig. 7), indicating that the role of *m*1/*m*2-type Trxs to regulate photosynthetic protein levels is important to support photosynthetic efficiency and CBC activity during HL acclimation. Although this indicates a role of Trxs *m*1/*m*2 in the acclimation response to prolonged growth under HL, they surprisingly play relatively minor roles in optimizing photosynthetic dynamics (Supplemental Fig. S1B and Fig. 2) and plant growth (Fig. 1A) under these conditions. This raises the hypothesis that long-term acclimation is not as important for plant growth as the rapid response to light changes.

### Trx *f*1 and trxs *m*1/*m*2 play different roles in light acclimation responses

Thioredoxins *f*1 *and f*2 are also part of the light-dependent Fdx-Trx system and comprise 22% of total chloroplast Trxs, with Trx *f*1 being the major *f*-type isoform (Okegawa and Motohashi 2015). In previous studies, Trx *f*1 has been found to play an important role in short-term activation of the CBC and starch synthesis during rapid dark-light transitions (Thormählen et al. 2013; Thormählen et al. 2015; Yoshida et al. 2015; Naranjo et al. 2016a). Here, we show that this does not extend to a substantial role of Trx *f*1 in light acclimation responses, indicating *f*-type Trxs to be involved in kick-starting photosynthesis during dark-light transitions but not in long-term acclimation responses to light intensity or variability. Unlike Trxs *m*1/*m*2 deficiencies, lack of Trx *f*1 did not lead to changes in acclimation responses at the proteome level in HL, while there were slight alterations in FL conditions (Fig. 8). This provides evidence for a minor contribution of Trx *f*1 to establish proteome changes in FL (Fig. 8), although this has no effects on photosynthesis, metabolite levels, ratios of CBC intermediates or plant growth (Figs. 1, 2, 6, and 8 and Supplemental Fig. S13). Interestingly, Trx *f*1 deficiency led to changes in the FBP/F6P and R5P/RuBP ratios in constant light conditions but not in FL, indicating a particular role of Trx *f*1 to activate FBPase and PRK of the CBC cycle in ML and LL (Fig. 6). This contrasts with Trxs *m*1/*m*2 deficiencies having no effects on CBC metabolite ratios. Overall, these findings indicate different roles of *f*- and *m*-type Trxs in light-acclimation responses.

### Remodeling of the proteome during HL acclimation is expected to involve NTRC- and Trxs-*m*1/*m*2-modulated retrograde signals

The results discussed above and summarized in Figs. 8 and 9 show that both NTRC and Trxs *m*1/*m*2 are important in triggering the remodeling of the proteome during HL acclimation. Since most proteins are encoded in the nucleus, and their expression involves nuclear transcription and cytosolic translation, this will involve retrograde signals to be modulated by these chloroplast thiol-redox regulators. It is, however, unclear how NTRC and Trxs *m*1/*m*2 are able to affect gene expression and translation beyond the chloroplast boundaries.

Chloroplast thiol dynamics can be linked to other compartments by the export of NAD(P)H-based redox equivalents (i.e. via triose-phosphate transport or malate/oxaloacetate shuttles) or by the release of H_2_O_2_ from the chloroplast (Zaffagnini et al. 2019; Meyer et al. 2021). However, the impact of these mechanisms to control redox processes beyond the chloroplast border in response to light changes remains to be clarified (Hebbelmann et al. 2012; Heyno et al. 2014). NTRC and Trxs *m*1/*m*2 were found to control the stromal NADP(H) redox poise and to promote redox-activation of NADP-MDH (Thormählen et al. 2017), a key enzyme of the malate valve to export reducing equivalents from the chloroplast (Scheibe 2004). The latter was proposed to be important under HL conditions (Heyno et al. 2014; Thormählen et al. 2017) and to optimize plant growth in FL intensities via its redox switch (Yokochi et al. 2021). In addition to this, NTRC was shown to modulate H_2_O_2_ levels by reducing 2-Cys Prxs in the chloroplast (Perez-Ruiz et al. 2006) and to regulate chloroplast-generated ROS involved in stress responses (Ishiga et al. 2016; Kim et al. 2017). By controlling the release of H_2_O_2_ from the chloroplast, NTRC may act on interorganellar redox signals to control the status of the cytosolic protein synthesis apparatus (Lokdarshi et al. 2020) and/or nuclear gene expression (Exposito-Rodriguez et al. 2017). Like NTRC, also *m*-type Trxs were found to modulate the level of ROS and the transcription of photosynthesis-related nuclear genes (Wang et al. 2013b). A possible role of NTRC in retrograde signaling from the chloroplast to the nucleus has also been suggested in previous transcriptomic studies (Lepistö et al. 2009; Lepistö et al. 2012), indicating NTRC to affect cytosolic protein quality control (Ojeda et al. 2021). This puts forward both NTRC and Trxs *m*1/*m*2 as modulators of retrograde signals in the acclimation to increasing light intensities, while Trx *f*1 is not involved in these responses.

## Conclusions

We discovered that HL, but surprisingly not FL, leads to large quantitative changes in the proteome and metabolome, resulting in increased photosynthetic dynamics and plant growth, suggesting light intensity is a stronger driver for acclimation than variability. It turned out that deficiencies in NTRC or Trxs *m*1/*m*2, but not Trx *f*1, almost completely suppressed the proteome remodeling which is required for long-term acclimation to light intensity, thus revealing previously unknown functions of both types of proteins in controlling dynamic light responses. While this suggests NTRC and Trxs *m*1/*m*2 to modulate (inter-)organellar signals to regulate cytosolic and chloroplast translation, the underlying mechanisms remain to be explored.

## Materials and methods

### Plant growth and harvesting

Arabidopsis (*Arabidopsis thaliana*) T-DNA insertion lines of Trx*f*1 (*trxf1*), Trx*m*1*m*2 (*trxm1.1m2.1*) and NTRC (*ntrc*) that have been characterized previously (Thormählen et al. 2017) were cultivated for 3 weeks in a LED chamber (12 h/12 h day/night, 60%/75% humidity day/night) with an initial illumination of 250 *µ*mol photons m^−2^ s^−1^ (ML). Following that, plants were shifted for an additional week either to HL (900 *µ*mol photons m^−2^ s^−1^), LL (90 *µ*mol photons m^–2^ s^–1^), or FL (1 min HL, 4 min LL; average light intensity 250 *µ*mol photons m^−2^ s^−1^) to analyze growth rates, photosynthetic performance, proteome and metabolome. For metabolic and proteomic analyses, plant rosettes were shock-frozen in liquid N_2_ under continuous illumination to immediately quench metabolism in the respective light conditions at 6 h into the photoperiod.

### Chlorophyll fluorescence and growth rate measurement

Analyses of acclimation kinetics: Measurements of chlorophyll a fluorescence (Fv/Fm) were performed at different time points after the shift in light conditions (see above) using a pulse-amplitude modulation (Imaging PAM, Walz, Germany) according to (Thormählen et al. 2017). Fv/Fm kinetics and changes in leaf areas were recorded during the 10-day growth phase in different light acclimation conditions 3 weeks after sowing, using ImageJ (https://imagej.nih.gov/ij; v.1.52a) and the following formula: A(t) = A × e ^ (λ × t). A = initial state, e = Euler's number, λ = growth constant, t = time.

Analyses of short-term dynamics: Measurements of chlorophyll a fluorescence in 4-week-old plants using a pulse-amplitude modulation (Imaging PAM, Walz, Germany). Minimal fluorescence yield (Fo) of dark-adapted (30 min) plants was measured before illumination with a short saturating pulse (2700 *µ*mol quanta m^−2^ s^−1^) to determine maximum quantum efficiency of PS II (Fv/Fm = (Fm-Fo)/Fm). Then, steady-state (Fs′) fluorescence was measured, followed by the application of short pulses of saturating light to determine maximal fluorescence (Fm′) and ground (Fo′) yield and in order to calculate PS II quantum yield (Φ II = (Fm′-Fs′)/Fm′), NPQ = (Fm-Fm′)/Fm′, and reduced PQ (1-qL =1—(((Fm′—Fs′) × Fo′)/((Fm′—Fo′) × Fs′))).

### Quantification of starch and protein levels

The extraction and determination of starch was performed according to previous publications using the pellets of the GC-MS extraction (Thormählen et al. 2013). For protein determination, desiccated pellets from the LC-MS/MS extraction were resuspended in 400 *µ*L 0.1 M NaOH, heated for 30 min, and quantified as published previously using BSA as standard (Bradford 1976).

### Metabolite profiling using GC-MS

Extraction of frozen plant material and subsequent analysis by gas chromatography coupled with mass spectrometry (GC-MS) was performed using the same equipment setup and protocol described previously (Lisec et al. 2006).

### Metabolite profiling using LC-MS/MS

After methanol/chloroform extraction of frozen plant material, metabolites were analyzed using an established reverse phase liquid chromatography coupled with tandem mass spectrometry (LC-MS/MS) platform (Arrivault et al. 2009). Stable isotopically labeled internal standards (SIL-IS) were added to rule out matrix effects for a subset of metabolites (Arrivault et al. 2015).

### Proteomic analysis

Proteins were prepared for mass spectrometric analysis by in-solution digestion using LysC and trypsin, and the resulting peptides were purified as described previously (Wiśniewski et al. 2009). Measurements were performed on a Q Exactive Plus HF mass spectrometer coupled with a nLC1000 nano-HPLC (both Thermofisher). Quantitative analysis was performed with MaxQuant v1.6.10.43 (https://maxquant.net/maxquant/) (Cox and Mann 2008) using standard settings. Peak lists were searched against the Arabidopsis reference proteome (UP000006548, April 2019). Data imputation was performed within Perseus v1.6.10.43 (https://maxquant.net/perseus/), where missing values were imputed with the default method for downstream processes that require a complete data set (Tyanova and Cox 2018). Data processing and functional analyses were done in R (R Core Team, 2021; https://www.R-project.org/), MapMan v3.5.1 and v3.6.0RC1 (https://mapman.gabipd.org/mapman) (Usadel et al. 2009) and Venny v2.1 (https://bioinfogp.cnb.csic.es/tools/venny/index.html) and using online databases Uniprot (https://www.uniprot.org/), agriGO (http://systemsbiology.cau.edu.cn/agriGOv2/; (Tian et al. 2017)), REVIGO (http://revigo.irb.hr/; (Supek et al. 2011)) and SUBA4 (https://suba.live/; (Hooper et al. 2017)). Quantitative correlation of photosynthetic protein levels and light intensity was adapted from KEGG pathways ath00190, ath00195 and ath00196 (https://www.genome.jp/kegg/). Raw data were deposited at PRIDE proteome exchange with identifier PXD045763 (https://www.ebi.ac.uk/pride/).

### Computational analyses and statistics

Under the assumption of a normal distribution, a one-factorial or a two-factorial analysis of variance (ANOVA) was performed as a standard test for parametric data with a following post hoc Tukey test. A repeated-measures *t*-test (reference WT or WT-ML) with a post hoc Benjamini–Hochberg correction (Benjamini and Hochberg 1995) served to evaluate proteomics. Testing and standardizing (z-score means) were carried out in R, using the “base”, “stats”, “emmeans” and “multcomp” packages (https://CRAN.R-project.org). Data wrangling was done with “tidyverse”, PCAs, heatmaps, violin- and boxplots were generated with “FactoMineR”, “factoextra”, “ComplexHeatmap” (https://bioconductor.github.io/BiocManager/) and “ggplot2”. Linear regression analysis of proteins (with FL) or metabolites (without FL) was done in Python with the “sklearn.linear_model” package (Pedregosa et al. 2011) (https://scikit-learn.org/) using light intensity as an independent variable. Coefficients were log10 transformed, where transformation of positive coefficients was multiplied by −1 while negative coefficients were multiplied by −1 prior to transformation (values closer to 1 or −1, respectively, indicate a stronger correlation with light intensity).

### Accession numbers

Arabidopsis Genome Initiative locus identifiers for the genes mentioned in this article are as follows: *NTRC* (AT2G41680), *TRXF1* (AT3G02730), *THM1* (AT1G03680), and *ATHM2* (AT4G03520).

## Supplementary Material

kiad535_Supplementary_Data

## Supplemental data

The following materials are available in the online version of this article.

**Supplemental Figure S1.** Rosette growth rates and photosynthetic dynamics as function of light intensity in WT trxf1, trxm1m2 and ntrc mutants acclimated to different light conditions.

**Supplemental Figure S2.** MapMan visualization of proteomic responses in different cell functions of the WT during acclimation to FL, HL or LL, relative to ML.

**Supplemental Figure S3.** MapMan visualization of proteomic responses in different cell functions of the ntrc mutant relative to wild-type during acclimation to FL, HL or LL conditions.

**Supplemental Figure S4.** MapMan visualization of proteomic responses in different cell functions of the trxm1m2 mutant relative to wild-type during acclimation to FL, HL or LL conditions.

**Supplemental Figure S5.** MapMan visualization of proteomic responses in different cell functions of the trxf1 mutant relative to wild-type during acclimation to FL, HL or LL conditions.

**Supplemental Figure S6.** MapMan visualization of proteomic responses involved in secondary metabolism and pigment synthesis in different genotypes during high-light acclimation.

**Supplemental Figure S7.** Overview of proteomic changes during acclimation to light intensity and variability in the WT.

**Supplemental Figure S8.** Overview of proteomic changes in trxf1, trxm1m2 and ntrc mutants relative to WT in different light conditions.

**Supplemental Figure S9.** Quantitative correlation of photosynthetic protein levels and light intensity in the WT and trxf1, trxm1m2 and ntrc mutants during light acclimation.

**Supplemental Figure S10.** Overview of light-dependent and genotypic dynamics in global metabolite levels.

**Supplemental Figure S11.** Overview of the dynamics in global metabolite levels in the WT and trxf1, trxm1m2 and ntrc mutants across different light intensities.

**Supplemental Figure S12.** Overview of light-dependent and genotypic dynamics in the CBC-related metabolite levels.

**Supplemental Figure S13.** Changes in the ratios of G6P/G1P and G1P/ADPG in WT, trxf1, trxm1m2 and ntrc mutants after acclimation to different light conditions.

**Supplemental Table S1.** Complete proteomic data set including mean values and statistics.

**Supplemental Table S2.** Significant functional categories of proteomic changes associated with high-light acclimation in the WT.

**Supplemental Table S3.** Significant functional categories of proteomic changes associated with low-light acclimation in the WT.

**Supplemental Table S4.** Significant functional categories of proteomic changes associated with fluctuating-light acclimation in the WT.

**Supplemental Table S5.** Significant functional categories of proteomic changes commonly associated with all light conditions (HL, LL, and FL) in the WT.

**Supplemental Table S6.** Significant functional categories of proteomic changes in the *ntrc* mutant compared to the WT under high-light acclimation.

**Supplemental Table S7.** Significant functional categories of common proteomic changes in the *ntrc* and *trxm1m2* mutants compared to the WT under high-light acclimation.

**Supplemental Table S8.** Core set of proteins with significant and common protein changes in *ntrc* and *trxm1m2* mutants during acclimation to HL, in comparison to the WT, focusing on oxidation–reduction processes or total.

**Supplemental Table S9.** Raw data of GC-MS based global metabolite profiles in WT, and *trxf1*, *trxm1m2* and *ntrc* mutants, acclimated to ML, FL, HL and LL.

**Supplemental Table S10.** Raw data of LC-MS/MS-based CBC-related metabolite profiles in the WT, and *trxf1*, *trxm1m2* and *ntrc* mutants, acclimated to ML, FL, HL and LL.

**Supplemental Table S11.** Number and subcellular localization of ribosomal proteins that were changed in their protein levels during acclimation to FL, HL or LL relative to ML in the WT.

**Supplemental Table S12.** Number of ribosomal proteins in chloroplast and cytosol that were changed in their protein levels during HL acclimation in the *ntrc* mutant relative to WT.

## Dive Curated Terms

The following phenotypic, genotypic, and functional terms are of significance to the work described in this paper:

trxf1 Gramene: AT3G02730
trxf1 Araport: AT3G02730

NADPH CHEBI: CHEBI:16474

ADP CHEBI: CHEBI:16761

pgr5 Gramene: AT2G05620
pgr5 Araport: AT2G05620

fructose CHEBI: CHEBI:28757
