# Supplementary material for: NTRC and thioredoxins m1/m2 underpin the light acclimation of plants on proteome and metabolome levels
Source: Plant Physiol. 2023 Oct 7;194(2):982–1005. doi: 10.1093/plphys/kiad535 (PMC10828201; doi:10.1093/plphys/kiad535)
Supplement: kiad535_Supplementary_Data [file kiad535_supplementary_data.zip › Suppl_Figs_Dejan_270623revised190923_edited final 031023n.pdf]

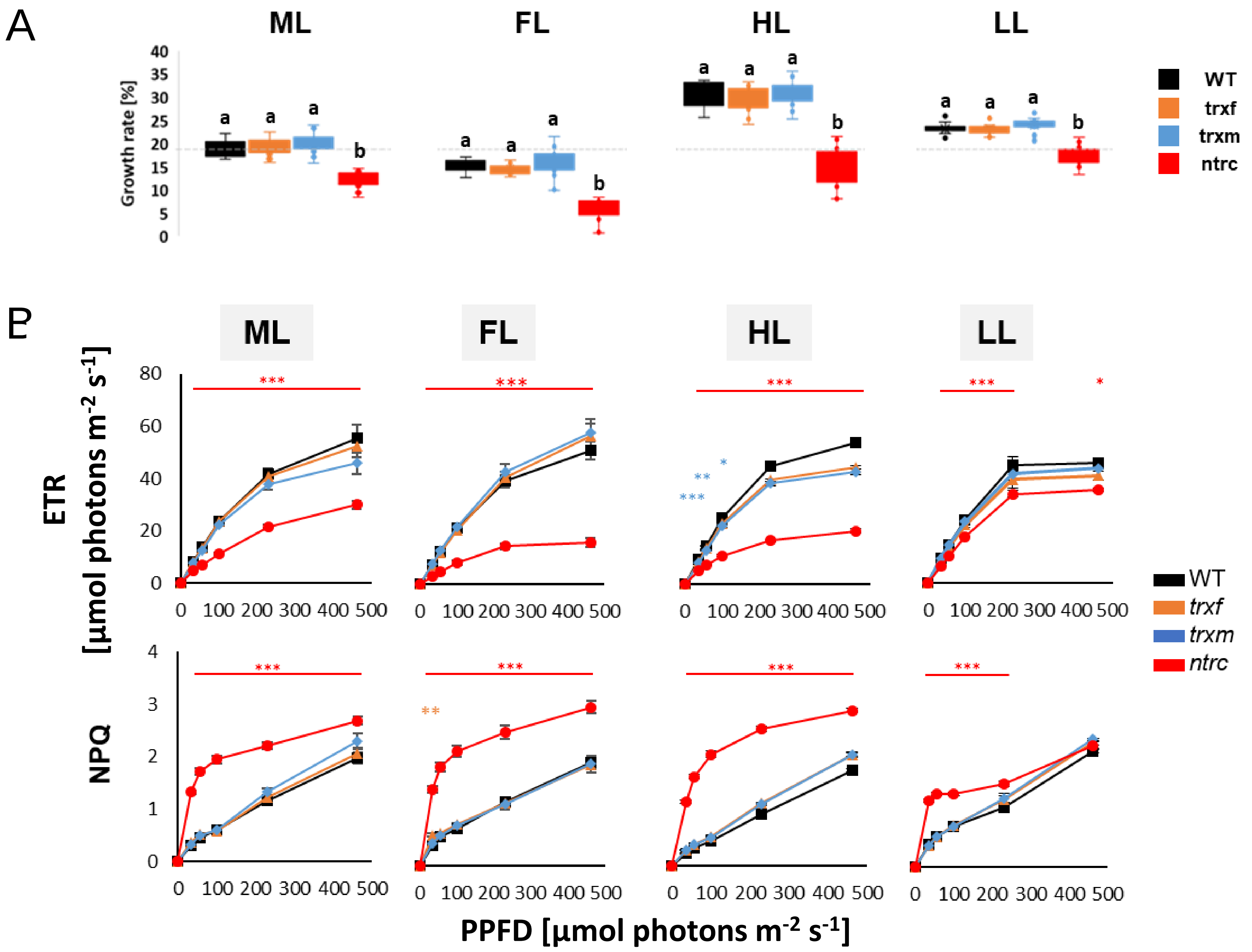
A
ML
FL
HL
LL
Growth rate [%]
WT
trxf
trxm
ntrc
B
ML
FL
HL
LL
ETR
[µmol photons m-2 s-1]
NPQ
PPFD [µmol photons m-2 s-1]

Figure S1: Rosette growth rates and photosynthetic dynamics as function of light intensity in wild type, *trxf1*, *trxm1m2* and *ntrc* mutants acclimated to different light conditions. Wild type (WT, black), *trxf1* (*trxf,* yellow), *trxm1m2* (*trxm,* blue) and *ntrc* (red) mutants were grown in 12h-day length at medium light intensity (ML, 250 µmol photons m^{-2} s^{-1}) for 3 weeks and then kept at this light intensity or shifted to either low light (LL, 90 µmol photons m^{-2} s^{-1}), high light (HL, 900 µmol photons m^{-2} s^{-1}) or fluctuating LL and HL (FL, 4 min LL, 1 min HL, average light intensity: ~250 µmol photons m^{-2} s^{-1}) to measure (**A**) rosette growth rates and (**B**) photosynthetic dynamics as function of light intensity. **(A)** Rosette-growth rates of the different genotypes after the shift to the respective light conditions are represented as boxplots. Measurements of rosette leave areas were performed every 2 days for 10 days total. The rate was calculated from curve fitting as mean value of all time points. The bottom and top of the boxplots are the 25th (Q1) and 75th (Q3) percentiles, respectively, and define the interquartile range (IQR; Q3-Q1). The whiskers extend to the maximum (Q3 + 1.5 * IQR) and the minimum (Q1 – 1.5 * IQR). Individual data points are symbolized by circles. The dashed line indicates the WT level in ML. Results are the mean, with n = 16-18 biological replicates per genotype and condition. Different letters indicate where the values of mutants are significantly different to the respective WT (one-way ANOVA with a *post-hoc* Tukey test; $p<0.05$). **(B)** Changes in electron transport and non-photochemical quenching (NPQ) as a function of short-term alterations in light intensity measured in the different genotypes 7 days after the shift to the respective light conditions. After dark-adaption (30 min) plants were subsequently exposed for 20 min to a step-wise increase in light intensities (six different light intensities ranging from 0 to 500 µE) to analyze time-resolved changes in chlorophyll a fluorescence to calculate electron transport rates (ETR = 0.5 × PPFD × Abs. × Φ II; top row) and NPQ (bottom row). Results are the mean ± SE, n = 5-12 biological replicates. Significant changes, relative to WT, within one time point were evaluated by using a two-way ANOVA with a *post-hoc* Tukey test (* $0.01 < p < 0.05$, ** $0.001 < p < 0.01$, *** $p < 0.001$). PPFD; photosynthetic photon flux density in µmol photons·m^{-2}·s^{-1}.

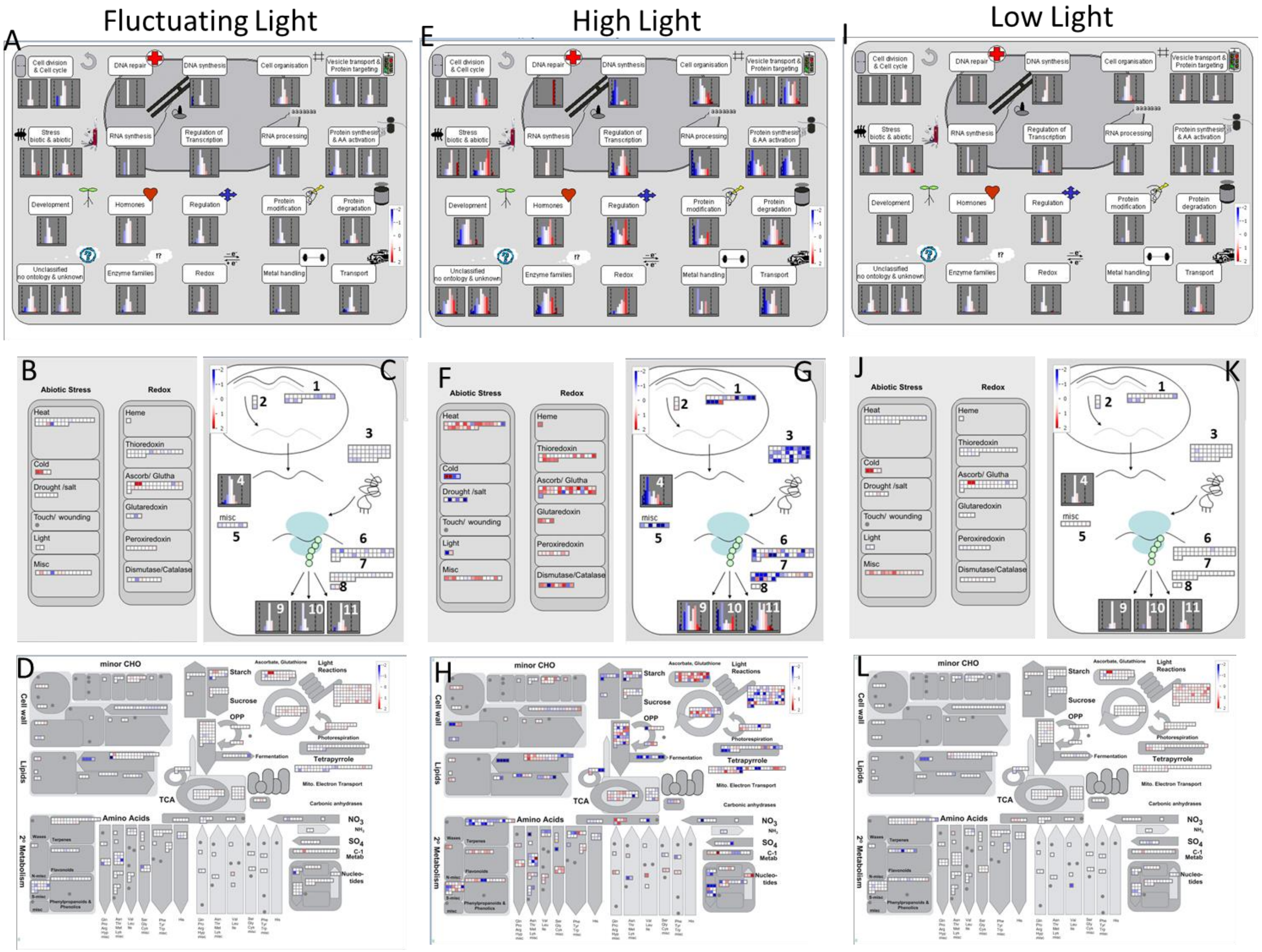

Figure S2: **MapMan visualisation of proteomic responses in different cell functions of the wild type during acclimation to fluctuating light, high light or low light, relative to medium light.** (**A**-**D**) Fluctuating light; (**E-H**) high light, (**I-L**) low light, with the following MapMan functional categories (**A, E, I**) cellular responses; (**B, F, J**) stress and redox; (**C, G, K**) gene expression, translation and protein metabolism, including 1) RNA processing, 2) RNA Transcription, 3) Amino acid activation, 4) Ribosomal protein, 5) Ribosome biogenesis, 6) Protein synthesis – initiation, 7) Protein synthesis – elongation, 8) Protein synthesis and -release 9) Protein targeting, 10) Post-translational modification, 11) Protein degradation; (**D, H, L**) metabolic overview. Relative abundances are shown as log2-fold changes ranging from -2 (blue) to 2 (red). White, no change. Graphs were generated with MapMan software. Analyses were performed 7 days after the light shift (see details in the legend to Figure S1).

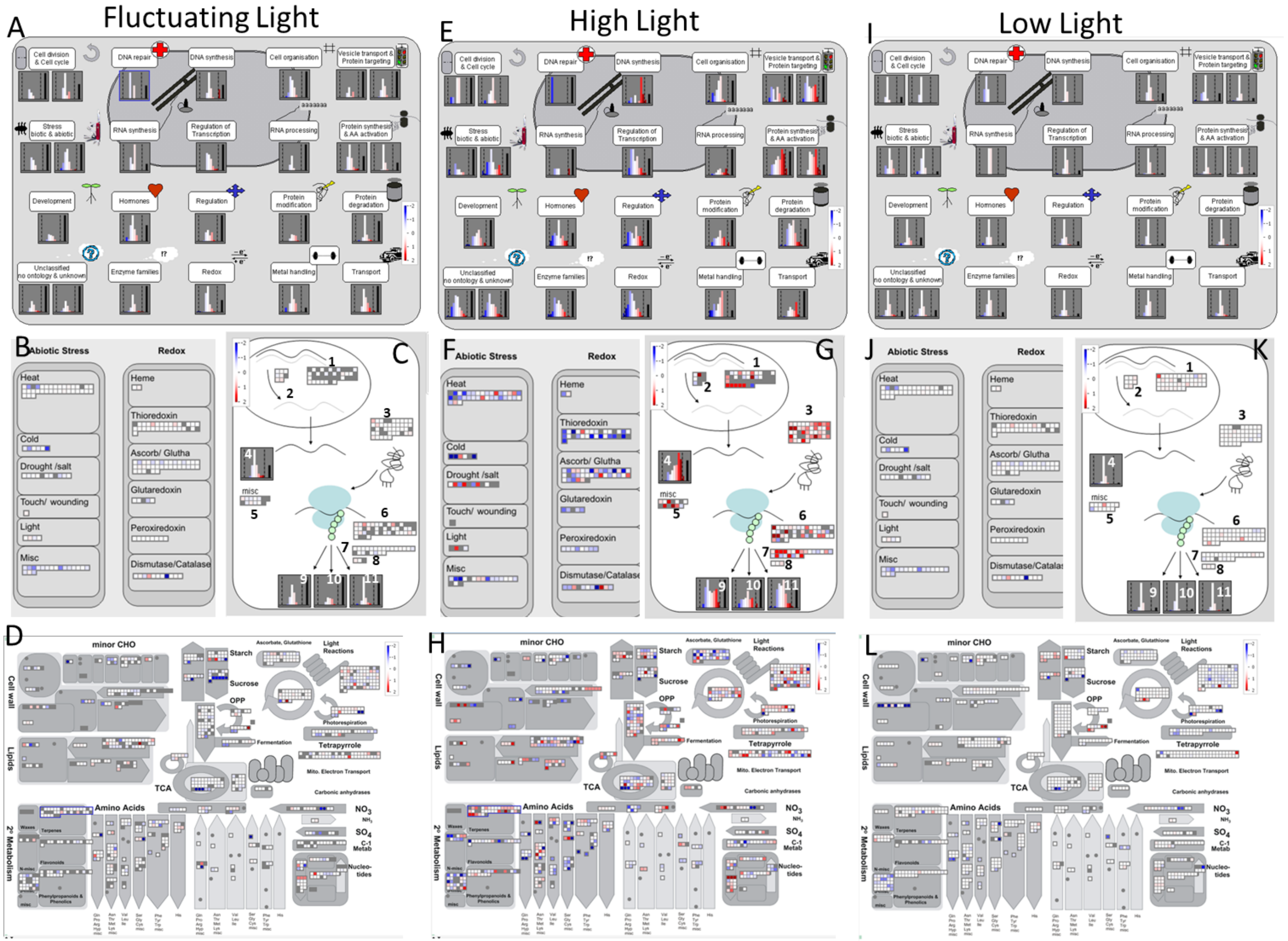

Figure S3: **MapMan visualisation of proteomic responses in different cell functions of the *ntrc* mutant relative to wild-type during acclimation to fluctuating light, high light or low light conditions.** (**A**-**D**) Fluctuating light; (**E-H**) high light, (**I-L**) low light, with the following MapMan functional categories (**A, E, I**) cellular responses; (**B, F, J**) stress and redox; (**C, G, K**) gene expression, translation and protein metabolism, including 1) RNA processing, 2) RNA Transcription, 3) Amino acid activation, 4) Ribosomal protein, 5) Ribosome biogenesis, 6) Protein synthesis – initiation, 7) Protein synthesis – elongation, 8) Protein synthesis and -release 9) Protein targeting, 10) Post-translational modification, 11) Protein degradation; (**D, H, L**) metabolic overview. Relative abundances are shown as log2-fold changes ranging from -2 (blue) to 2 (red). White, no change. Graphs were generated with MapMan software. Analyses were performed 7 days after the light shift (see details in the legend to Figure S1)..

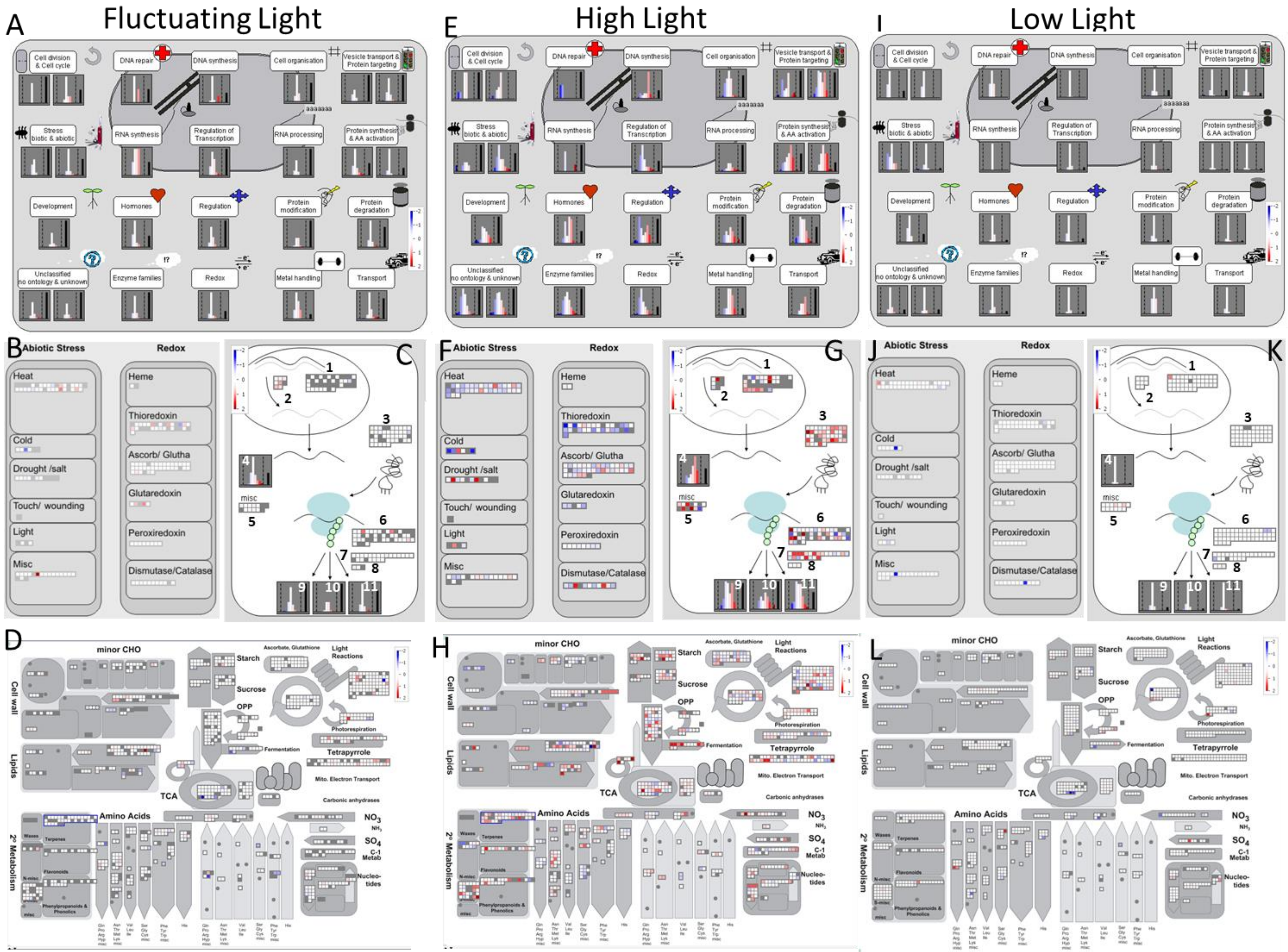

Figure S4: **MapMan visualisation of proteomic responses in different cell functions of the *trxm1m2* mutant relative to wild-type during acclimation to fluctuating light, high light or low light conditions.** (**A**-**D**) Fluctuating light; (**E-H**) high light, (**I-L**) low light, with the following MapMan functional categories (**A, E, I**) cellular responses; (**B, F, J**) stress and redox; (**C, G, K**) gene expression, translation and protein metabolism, including 1) RNA processing, 2) RNA Transcription, 3) Amino acid activation, 4) Ribosomal protein, 5) Ribosome biogenesis, 6) Protein synthesis – initiation, 7) Protein synthesis – elongation, 8) Protein synthesis and -release 9) Protein targeting, 10) Post-translational modification, 11) Protein degradation; (**D, H, L**) metabolic overview. Relative abundances are shown as log2-fold changes ranging from -2 (blue) to 2 (red). White, no change. Graphs were generated with MapMan software. Analyses were performed 7 days after the light shift (see details in the legend to Figure S1).

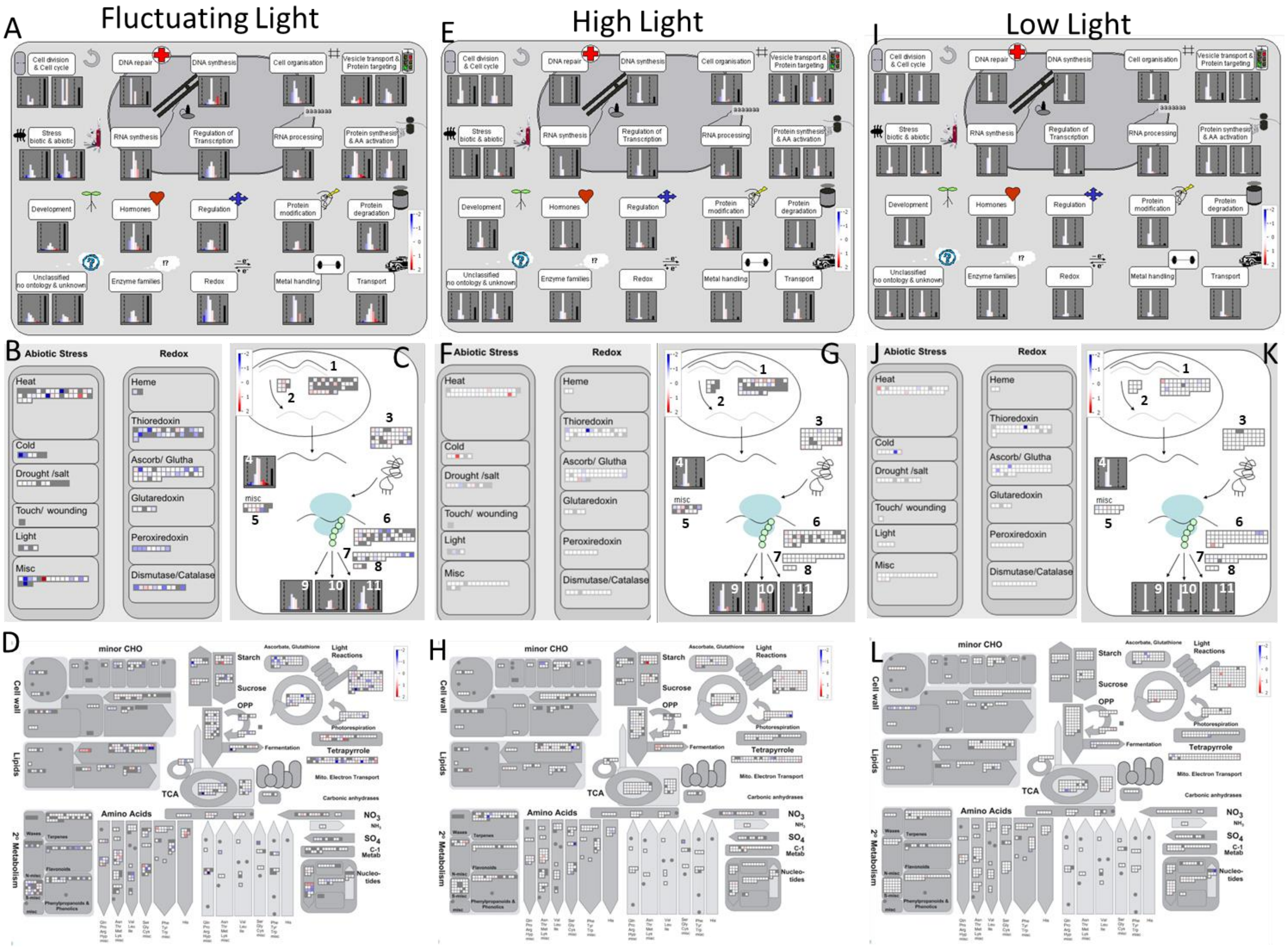

Figure S5: **MapMan visualisation of proteomic responses in different cell functions of the *trxf1* mutant relative to wild-type during acclimation to fluctuating light, high light or low light conditions.** (**A**-**D**) Fluctuating light; (**E-H**) high light, (**I-L**) low light, with the following MapMan functional categories (**A, E, I**) cellular responses; (**B, F, J**) stress and redox; (**C, G, K**) gene expression, translation and protein metabolism, including 1) RNA processing, 2) RNA Transcription, 3) Amino acid activation, 4) Ribosomal protein, 5) Ribosome biogenesis, 6) Protein synthesis – initiation, 7) Protein synthesis – elongation, 8) Protein synthesis and -release 9) Protein targeting, 10) Post-translational modification, 11) Protein degradation; (**D, H, L**) metabolic overview. Relative abundances are shown as log2-fold changes ranging from -2 (blue) to 2 (red). White, no change. Graphs were generated with MapMan software. Analyses were performed 7 days after the light shift (see details in the legend to Figure S1).

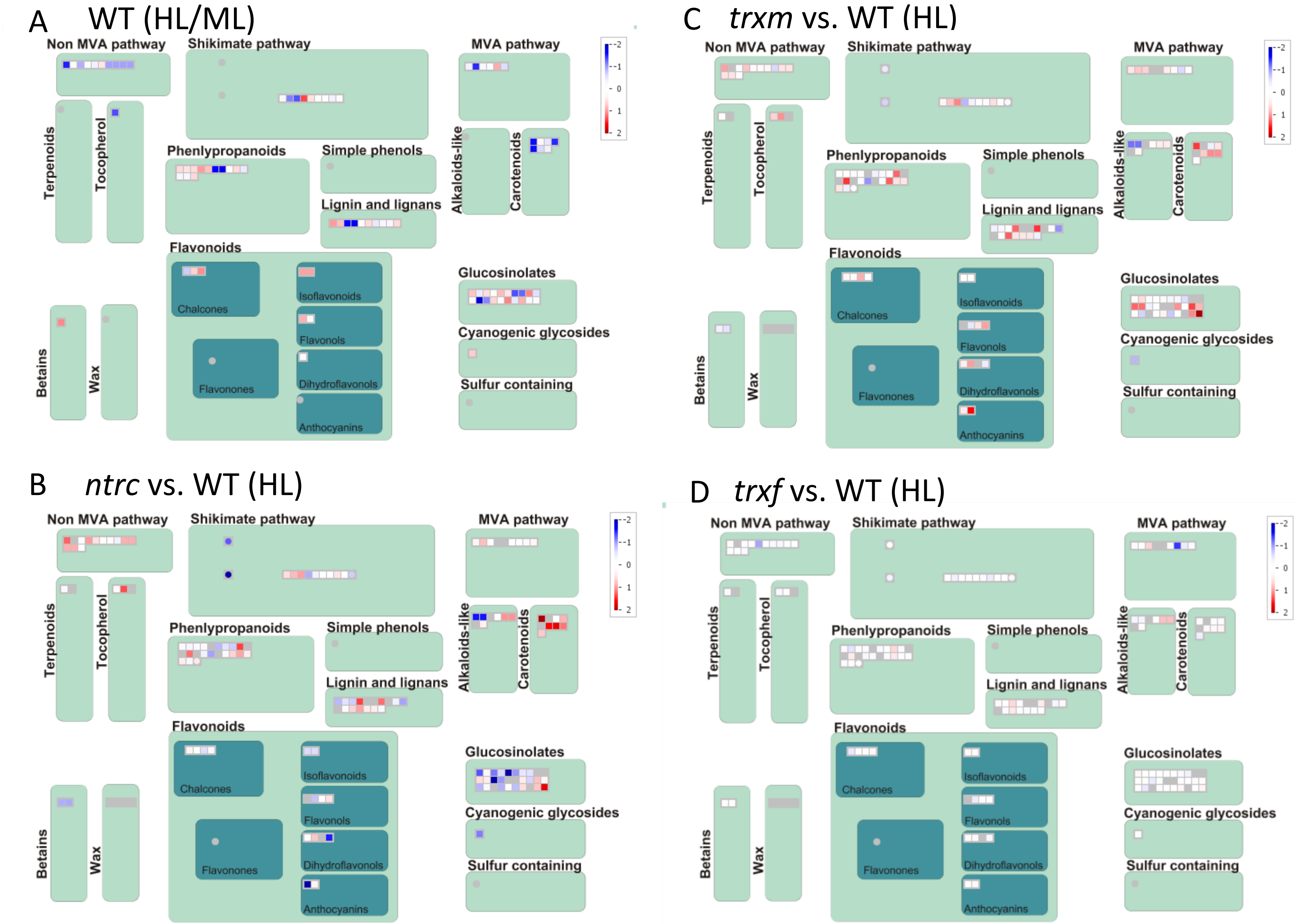

Figure S6: **MapMan visualisation of proteomic responses involved in secondary metabolism and pigment synthesis in different genotypes during high-light acclimation** (**A**) Wild type (WT) responses in HL vs. ML; (**B**) *ntrc* mutant responses vs. WT in HL; (**C**) *trxm1m2 (trxm)* mutant responses vs. WT in HL; (D) *trxf1 (trxf)* mutant responses vs. WT in HL. Relative abundances are shown as log2-fold changes ranging from -2 (blue) to 2 (red). White, no change. Graphs were generated with MapMan software. Analyses were performed 7 days after the light shift (see details in the legend to Figure S1).

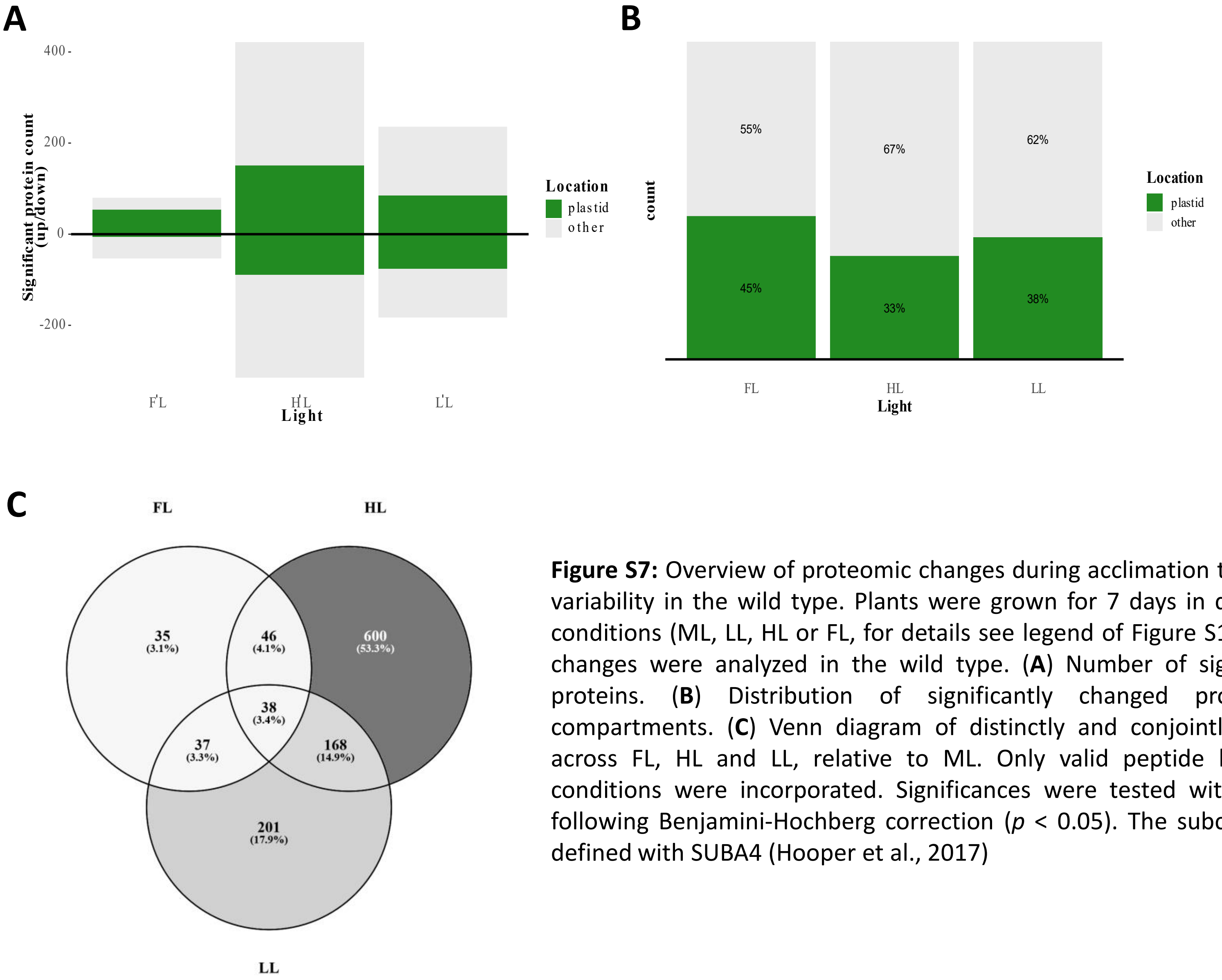

Figure S7: Overview of proteomic changes during acclimation to light intensity and variability in the wild type. Plants were grown for 7 days in different acclimation conditions (ML, LL, HL or FL, for details see legend of Figure S1), before proteomic changes were analyzed in the wild type. (**A**) Number of significantly regulated proteins. (**B**) Distribution of significantly changed proteins across cell compartments. (**C**) Venn diagram of distinctly and conjointly changed proteins across FL, HL and LL, relative to ML. Only valid peptide hits common in all conditions were incorporated. Significances were tested with a repeated t-test following Benjamini-Hochberg correction ($p < 0.05$). The subcellular location was defined with SUBA4 (Hooper et al., 2017)

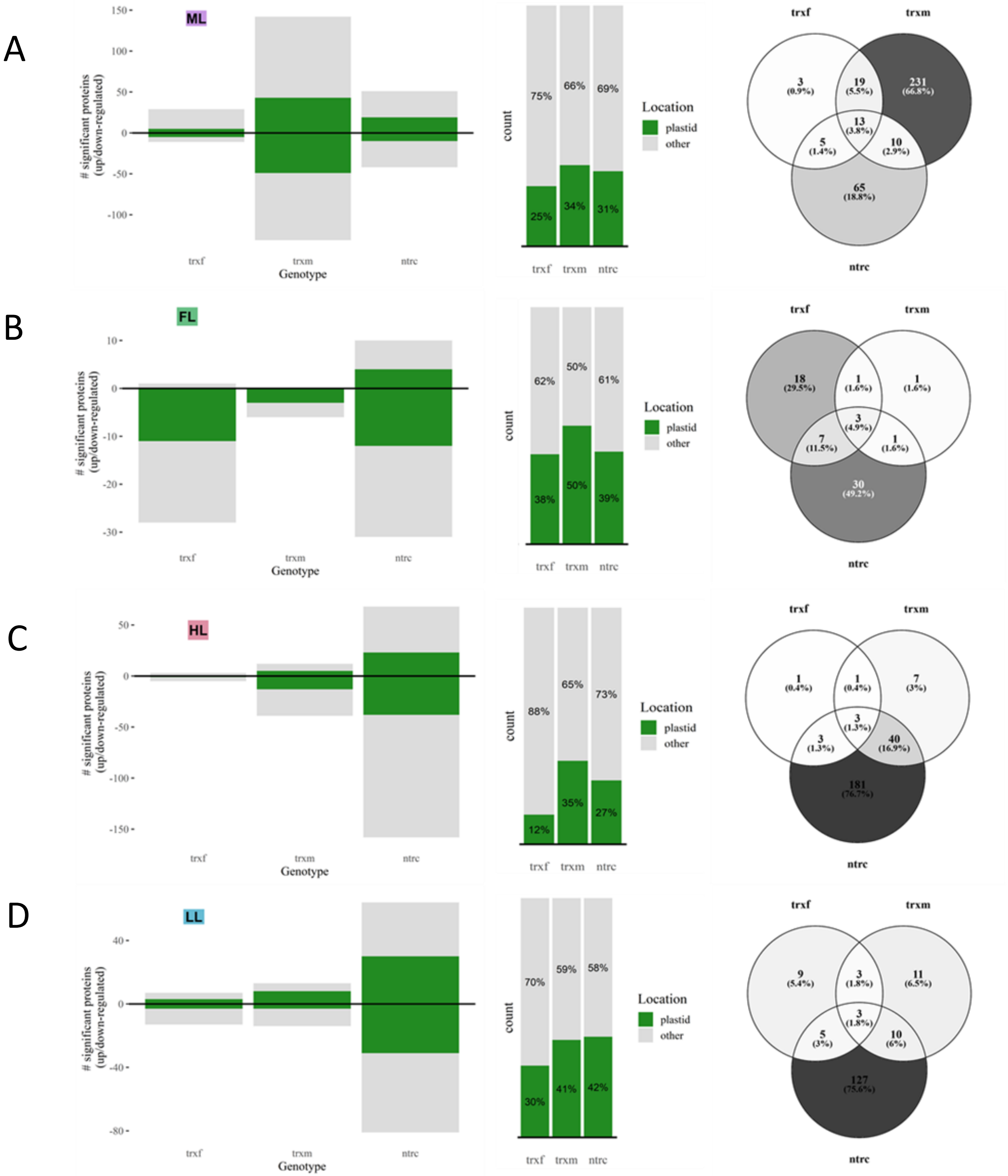

Figure S8: **Overview of proteomic changes in *trxf1, trxm1m2 and ntrc* mutants relative to wild type in different light conditions.** Plants were grown for 7 days in different acclimation conditions (ML, LL, HL or FL, for details see legend of Figure S1), before proteomic changes were analyzed in the mutants relative to wild type. Summary and distribution of significantly up- or down-regulated proteins across cell compartments and Venn diagrams of commonly and exclusively changed proteins in (**A**) ML, (**B**) FL, (**C**) HL and (**D**) LL. Significances were tested with a repeated *t*-test following Benjamini-Hochberg correction ($p < 0.05$). The subcellular location was defined with SUBA4 (Hooper et al., 2017).

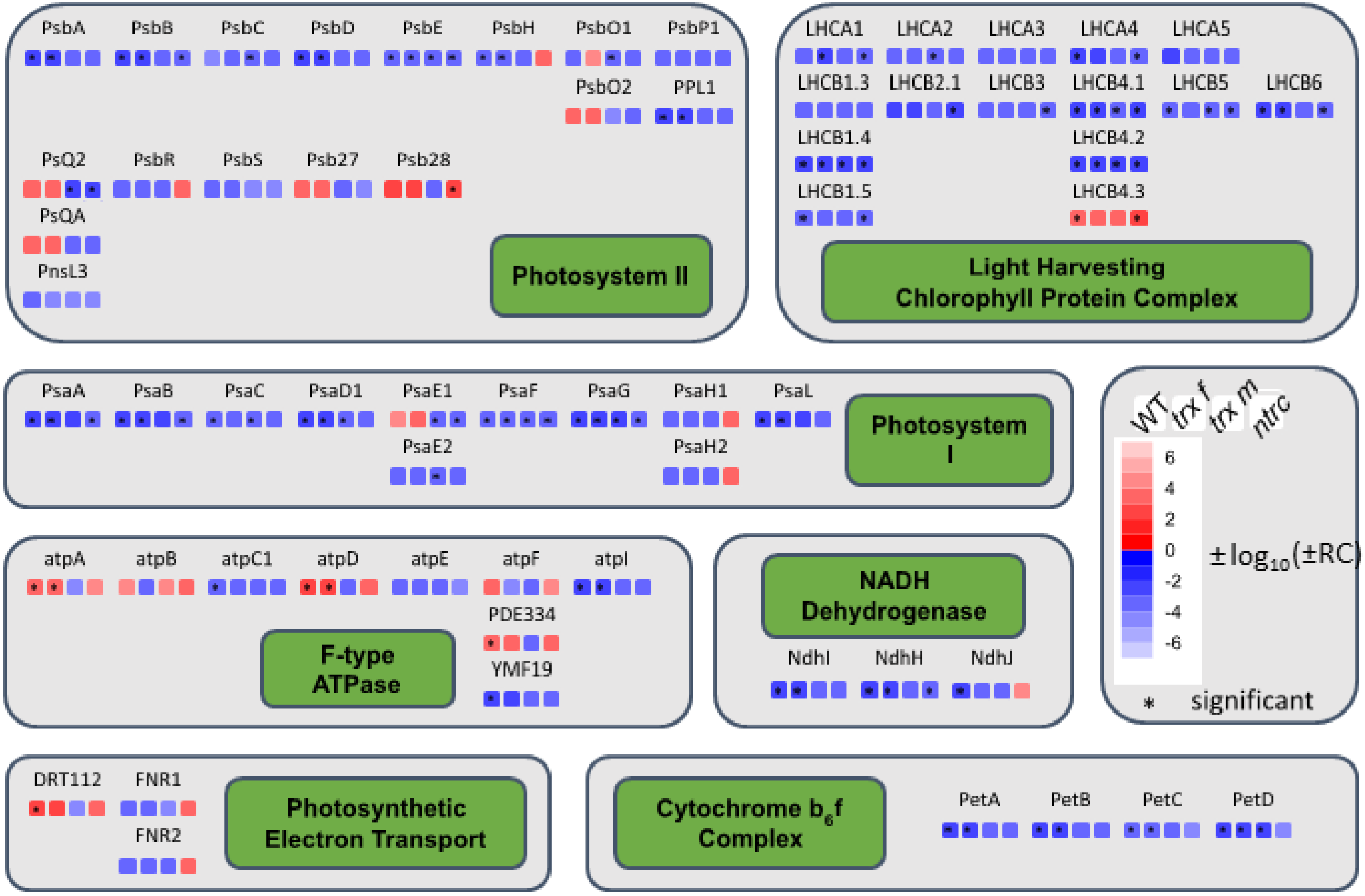

Figure S9: **Quantitative correlation of photosynthetic protein levels and light intensity in wild type, *trxf1*, *trxm1m2* and *ntrc* mutants during light acclimation.** Linear regression analysis of photosynthetic proteins was performed using acclimation-light intensity (photon flux density in µE) as independent variable. The heat map shows log10-transformed regression coefficients, where transformation of positive coefficients was multiplied by -1 while negative coefficients were multiplied by -1 prior to transformation (values closer to 1 or -1, respectively, indicate a stronger correlation with light intensity). Significance levels ($p < 0.05$) are labelled with asterisks. RC, regression coefficient; WT, wild type; trxf, *trxf1;* trxm, *trxm1m2; ntrc* (ntrc).

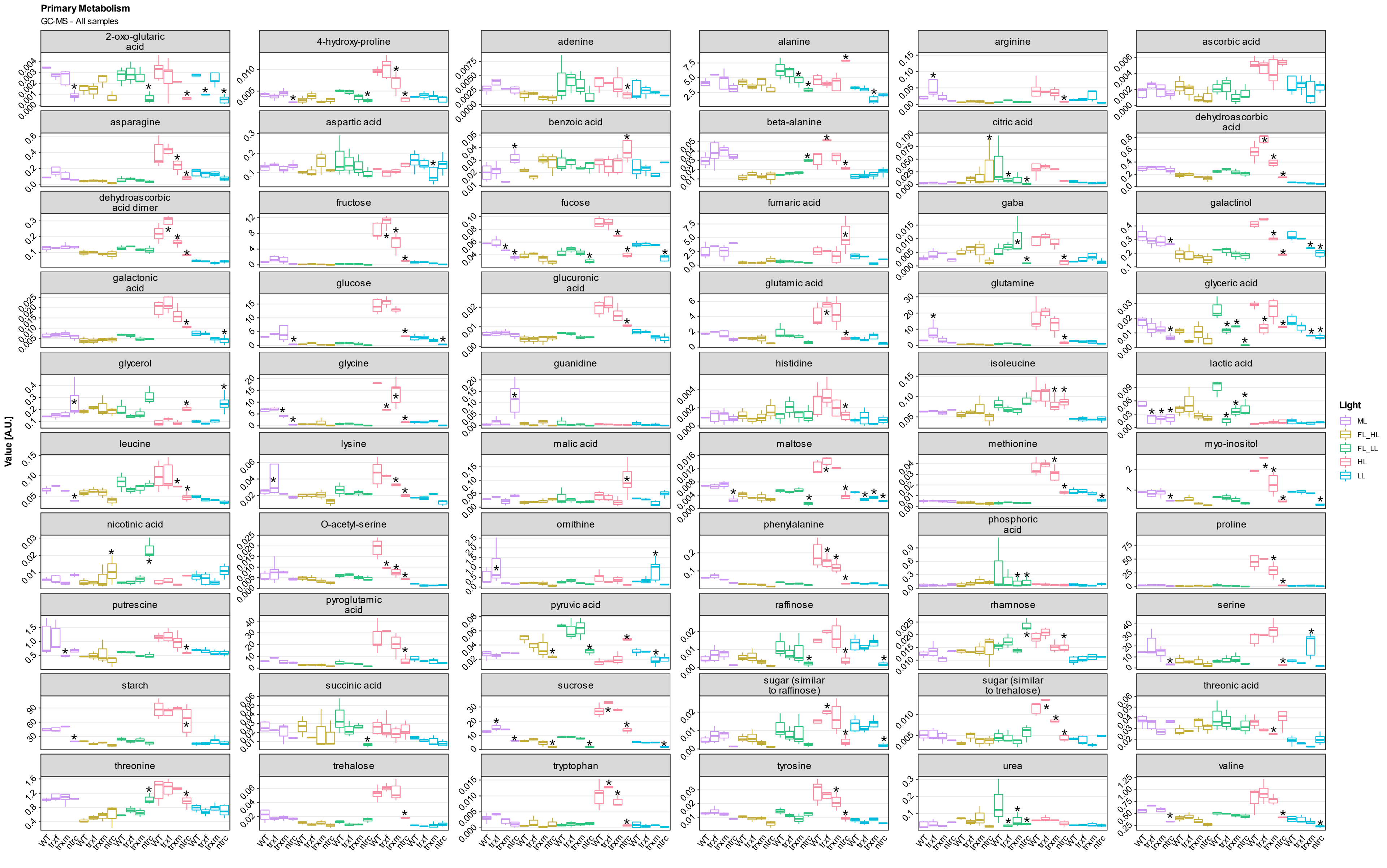

Figure S10: Overview of light-dependent and genotypic dynamics in global metabolite levels. Wild type (WT), *trxf1* (trxf), *trxm1m2* (trxm) and *ntrc* (ntrc) mutants were grown for 7 days in different light acclimation conditions (ML, LL, HL or FL, for details see legend of Figure S1), before the GC-MS based global metabolome was analyzed. Results are shown as boxplots in arbitrary units, *n* = 3-5 biological replicates. The line in the middle of the boxplots represents the median. The bottom and top of the boxplots are the 25th (Q1) and 75th (Q3) percentiles, respectively, and define the interquartile range (IQR; Q3-Q1). The whiskers extend to the maximum (Q3 + 1.5 * IQR) and the minimum (Q1 – 1.5 * IQR) without outliers. Asterisks indicate where the values of mutants are significantly different to the respective WT (ANOVA with a post-hoc Tukey test; p<0.05) ML, medium light (pink); FL HL, HL phase of FL (brown); FL LL, LL phase of FL (green); HL, high light (red); LL, low light (blue). Raw data, see Table S9.

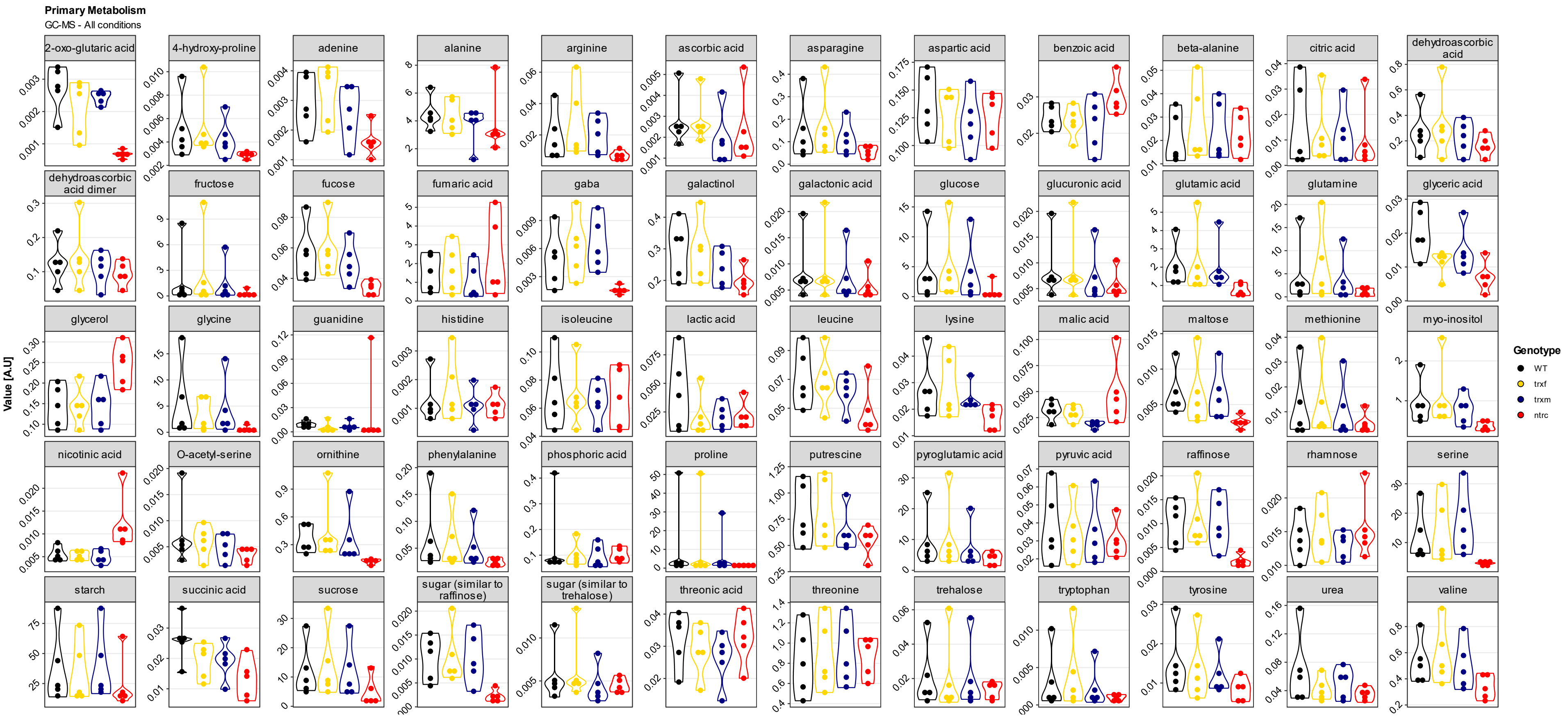

Figure S11: Overview of the dynamics in global metabolite levels in in wild type, *trxf1*, *trxm1m2* and *ntrc* mutants across different light intensities. Wild type (WT, black), *trxf1* (trxf, yellow), *trxm1m2* (trxm, blue) and *ntrc* (ntrc, red) mutants were shifted for 7 days into different acclimation conditions (ML, LL, HL or FL, for details see legend of Figure S1), before changes in the GC-MS based global metabolome were analyzed. The violin plots show the pooled mean values of GC-MS based metabolite levels across all light conditions (ML, FL HL, FL LL, HL, LL, for details see legend to Figure S10). Values are in arbitrary units. Raw data, see Table S9.

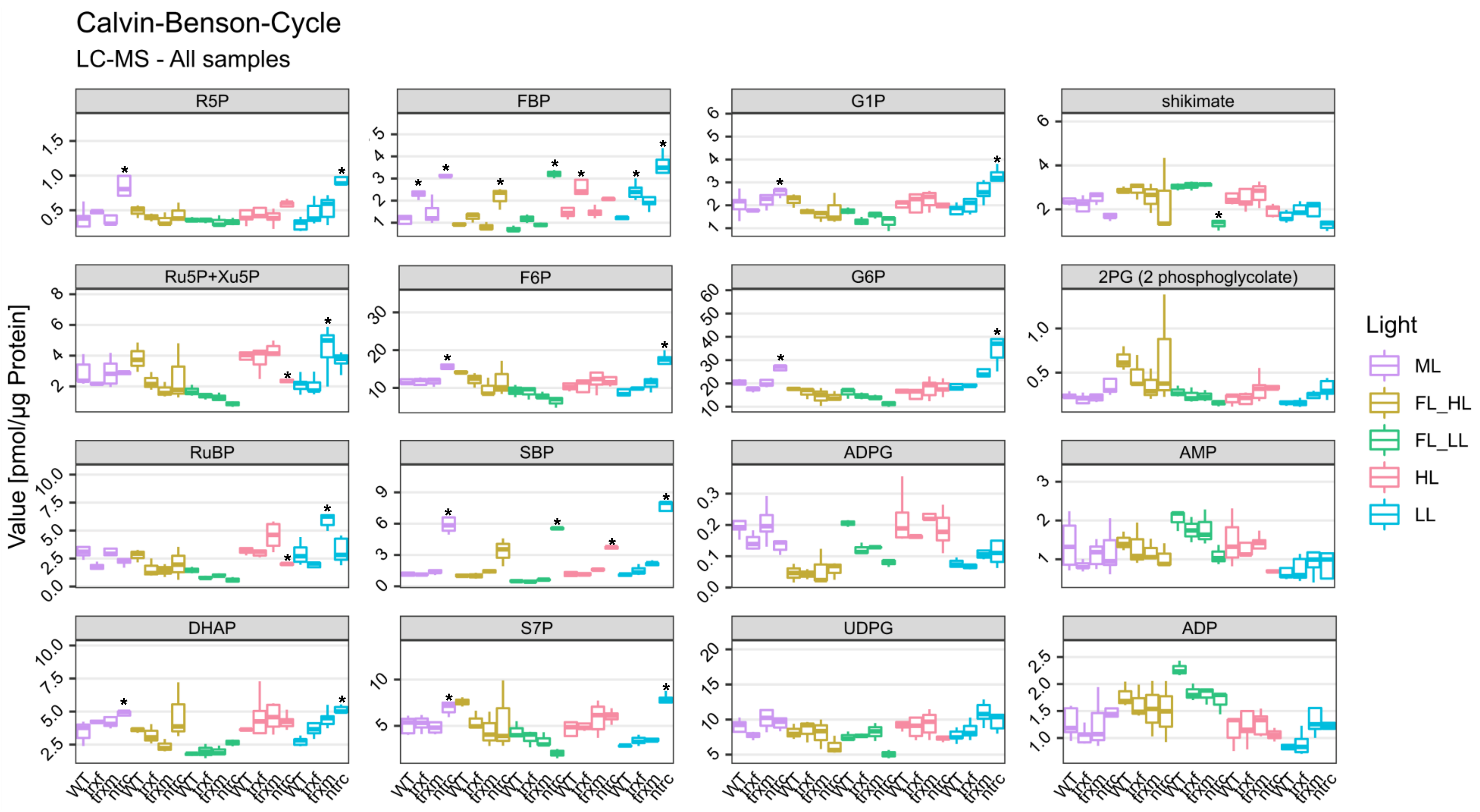

Figure S12: Overview of light-dependent and genotypic dynamics in Calvin-Benson-cycle related metabolite levels. Wild type (WT), *trxf1* (trxf), *trxm1m2* (trxm) and *ntrc* (ntrc) mutants were grown for 7 days in different light acclimation conditions (ML, LL, HL or FL, for details see legend of Figure S1), before the LC-MS/MS based Calvin-Benson-cycle related metabolome was analyzed. Results are shown as boxplots in pmol/µg protein, *n*=3-5 biological replicates. The line in the middle of the boxplots represents the median. The bottom and top of the boxplots are the 25th (Q1) and 75th (Q3) percentiles, respectively, and define the interquartile range (IQR; Q3-Q1). The whiskers extend to the maximum (Q3 + 1.5 * IQR) and the minimum (Q1 – 1.5 * IQR) without outliers. Asterisks indicate where the values of mutants are significantly different to the respective WT (ANOVA with a post-hoc Tukey test; $p<0.05$) . ML, medium light (pink); FL HL, HL phase of FL (brown); FL LL, LL phase of FL (green); HL, high light (red); LL, low light (blue). Raw data, see Table S10.

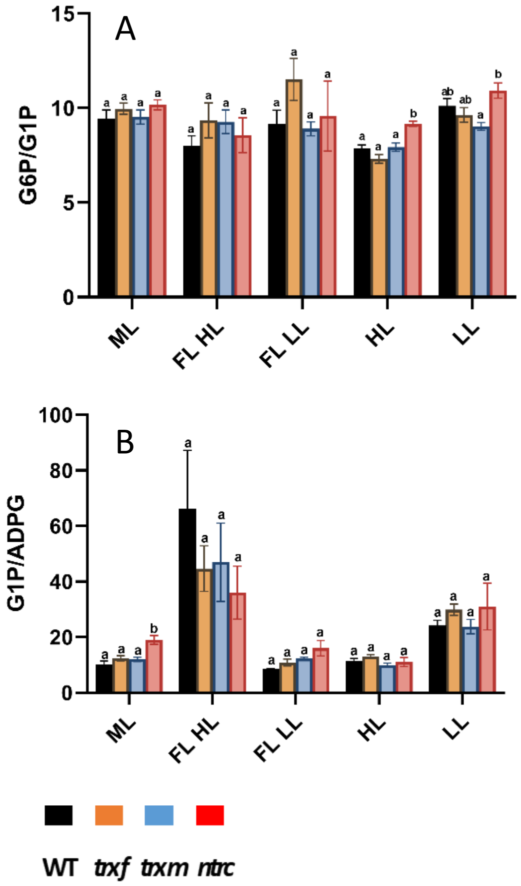

Figure S13: Changes in the ratios of G6P/G1P and G1P/ADPG in wild type, *trxf1*, *trxm1m2* and *ntrc* mutants after acclimation to different light conditions. Wild type (WT, black), *trxf1* (*trxf*, yellow), *trxm1m2* (*trxm*, blue) and *ntrc* (*ntrc*, red) mutants were shifted for 7 days into different acclimation conditions (ML, LL, HL or FL, for details see legend of Figure S1), before changes in the levels of LC-MS/MS based Calvin-Benson-cycle related metabolites were analyzed to calculate metabolite ratios. (**A**) G6P/G1P. (**B**) G1P/ADPG. Results are the mean ± SE, *n* = 3-5 biological replicates. Significance levels within one condition were evaluated by using a one-way ANOVA with a *post-hoc* Tukey test ($p < 0.05$) and are labelled with different letters. ML, medium light; FL LL, LL phase of FL; FL HL, HL phase of FL, HL, high light; LL, low light
